# Proportional odds ratio model for comparison of diagnostic tests in meta-analysis

**DOI:** 10.1186/1471-2288-4-27

**Published:** 2004-12-10

**Authors:** Mir Said Siadaty, Jianfen Shu

**Affiliations:** 1 Division of Biostatistics and Epidemiology, University of Virginia School of Medicine, Box 800717, Charlottesville, Virginia, 22908, USA

## Abstract

**Background:**

Consider a meta-analysis where a 'head-to-head' comparison of diagnostic tests for a disease of interest is intended. Assume there are two or more tests available for the disease, where each test has been studied in one or more papers. Some of the papers may have studied more than one test, hence the results are not independent. Also the collection of tests studied may change from one paper to the other, hence incomplete matched groups.

**Methods:**

We propose a model, the proportional odds ratio (POR) model, which makes no assumptions about the shape of *OR_p_*, a baseline function capturing the way OR changes across papers. The POR model does not assume homogeneity of ORs, but merely specifies a relationship between the ORs of the two tests.

One may expand the domain of the POR model to cover dependent studies, multiple outcomes, multiple thresholds, multi-category or continuous tests, and individual-level data.

**Results:**

In the paper we demonstrate how to formulate the model for a few real examples, and how to use widely available or popular statistical software (like SAS, R or S-Plus, and Stata) to fit the models, and estimate the discrimination accuracy of tests. Furthermore, we provide code for converting ORs into other measures of test performance like predictive values, post-test probabilities, and likelihood ratios, under mild conditions. Also we provide code to convert numerical results into graphical ones, like forest plots, heterogeneous ROC curves, and post test probability difference graphs.

**Conclusions:**

The flexibility of POR model, coupled with ease with which it can be estimated in familiar software, suits the daily practice of meta-analysis and improves clinical decision-making.

## Background

A diagnostic test, in its simple form, tries to detect presence of a particular condition (disease) in a sample. Usually there are several studies where performance of the diagnostic test is measured by some statistic. One may want to combine such studies to get a good picture of performance of the test, a meta-analysis. Also, for a particular disease there may be several diagnostic tests invented, where each of the tests is subject of one or more studies. One may also want to combine all such studies to see how the competing tests are performing with respect to each other, and choose the best for clinical practice.

To pool several studies and estimate a summary statistic some assumptions are made. One such assumption is that differences seen between individual study results are due to chance (sampling variation). Equivalently, this means all study results are reflecting the same "true" effect [1]. However, meta-analysis of studies for some diagnostic tests show that this assumption, in some cases, is not empirically supported. In other words, there is more variation between the studies that could be explained by random chance alone, the so-called "conflicting reports". One solution is to relax the assumption that every study is pointing to the same value. In other words, one accepts explicitly that different studies may correctly give "different" values for performance of the same test.

For example, sensitivity and specificity are a pair of statistics that together measure the performance of a diagnostic test. One may want to compute an average sensitivity and an average specificity for the test across the studies, hence pooling the studies together. Instead, one may choose to extract odds ratio (OR) from each paper (as test performance measure), and then estimate the average OR across the studies. The advantage is that widely different sensitivities (and specificities) can point to the same OR. This means one is relaxing the assumption that all the studies are pointing to the same sensitivity and specificity, and accepts that different studies are reporting "truly different" sensitivity and specificity, and that the between-study variation of them is not due to random noise alone, but because of difference in choice of decision threshold (the cutoff value to dichotomize the results). Therefore the major advantage of OR, and its corresponding receiver-operating-characteristic (ROC) curve, is that it provides measures of diagnostic accuracy unconfounded by decision criteria [2]. An additional problem when pooling sensitivities and specificities separately is that it usually underestimates the test performance [[3], p.670].

The above process may be used once more to relax the assumption that every study is pointing to the same OR, thus relaxing the "OR-homogeneity" assumption. In other words, in some cases, the remaining variation between studies, after utilizing OR as the summary performance measure, is still too much to be attributed to random noise. This suggests OR may vary from study to study. Therefore one explicitly assumes different studies are measuring different ORs, and that they are not pointing to the same OR. This difference in test performance across studies may be due to differences in study design, patient population, case difficulty, type of equipment, abilities of raters, and dependence of OR on threshold chosen [4]. Nelson [5] explains generating ROC curves that allow for the possibility of "inconstant discrimination accuracy", a heterogeneous ROC curve (HetROC). This means the ROC curve represents different ORs at different points. This contrasts with the fact that the homogeneous-ROC is completely characterized by one single OR.

There are a few implementations of the heterogeneous ROC. One may classify them into two groups. The first group is exemplified by Tosteson and Begg [6]. They show how to use ordinal regression with two equations that correspond to location and scale. The latent scale binary logistic regression of Rutter and Gatsonis [4] belong to this group. The second group contains implementations of Kardaun and Kardaun [7], and Moses et al [8]. Moses et al explain a method to plot such heterogeneous ROC curve under some parametric assumptions, and they call it summary ROC (SROC).

When comparing two (or more) diagnostic tests, where each study reports results on more than one test, the performance statistics (in the study results) are correlated. Then standard errors computed by SROC are invalid. Toledano and Gatsonis [9] use the ordinal regression model, and account for the dependency of measurements by generalized estimating equations (GEE). However, to fit the model they suggest using a FORTRAN code.

We propose a regression model that accommodates more general heterogeneous ROC curves than SROC. The model accommodates complex missing patterns, and accounts for correlated results [10]. Furthermore, we show how to implement the model using widely available statistical software packages. The model relaxes OR-homogeneity assumption. In the model, when comparing two (or more) tests, each test has its own trend of ORs across studies, while the trends of two tests are (assumed to be) proportional to each other, the "proportional odds ratio" assumption. We alleviate dilemma of choosing weighting schemes such that do not bias the estimates [[11], p.123], by fitting the POR model to 2-by-2 tables. The model assumes a binomial distribution that is more realistic than a Gaussian used by some implementations of HetROC. Also, it is fairly easy to fit the model to (original) patient level data (if available).

Besides accounting better for between-study variation, we show how to use the POR model to "explain why" such variation exists. This potentially gives valuable insights and may have direct clinical applications. It may help define as to when, where, how, and on what patient population to use which test, to optimize performance.

We show how to use "deviation" contrast, in parameterization of categorical variables, to relax the restriction that a summary measure may be reported only if the respective interaction terms in the model are insignificant. This is similar to using grand mean in a "factor effects" ANOVA model (compared to "cell means" ANOVA model).

We show how to use nonparametric smoothers, instead of parametric functions of true positive rate (TPR) and/or false positive rate (FPR), to generate heterogeneous ROC for a single diagnostic test across several studies.

Our proposed POR model assumes the shape of the heterogeneous ROC curve is the same from one test to the other, but they differ in their locations in the ROC space. This assumption facilitates the comparison of the tests. However, one may want to relax the POR assumption, where each test is allowed to have a heterogeneous ROC curve with a different shape. One may implement such generalized comparison of the competing diagnostic tests by a mixed effects model. This may improve generalizability of meta-analysis results to all (unobserved) studies. Also, a mixed effects model may take care of remaining between-study variation better.

## Methods

### Average difference in performances

To compare two diagnostic tests i and j, we want to estimate the difference in their performance. However, in reality such difference may vary from one paper (study) to the other. Therefore Δ_i,j,p _= PERF_i,p _- PERF_j,p_, where the difference Δ depends on paper index p, where PERF_i,p _is observed performance of test i in paper p. To simplify notation, assume that a single number measures performance of each test in each paper. We relax this assumption later, allowing for the distinction between the two types of mistakes (FNR and FPR, or equivalently TPR and FPR). We decompose the differences

*(1) *Δ_*i,j,p *_= *PERF_i,p _*- *PERF_j,p_*= *δ_i,j _*+ *δ_i,j,p_*,

where *δ*_i,j _is the 'average' difference between the two tests, and *δ*_i,j,p _is deviation of the observed difference within paper p from the average *δ*_i,j_. The *δ*_i,j _is an estimator for the difference between performance of the two tests. Note by using deviation parameterization (similar to an ANOVA model) [[12], pp.51 & 45] we explicitly accept and account for the fact that the observed difference varies from one paper to the other, while estimating the 'average' difference. This is similar to a random-effects approach where a random distribution is assumed for the Δ_i,j,p _and then the mean parameter for the distribution is estimated. In other words, one does not need to assume 'homogeneous' difference of the two tests across all the papers, and then estimate the 'common' difference [13].

The observed test performance, PERF, may be measured in several different scales, such as paired measures sensitivity and specificity, positive and negative predictive values, likelihood ratios, post test odds, and post test probabilities for normal and abnormal test results; as well as single measures such as accuracy, risk or rate ratio or difference, Youden's index, area under ROC curve, and odds ratio (OR). When using OR as the performance measure, the marginal logistic regression model

*(2) logit(Result_pt_) *= *β_0 _*+ β_1_*Disease_pt _+ β_2_*PaperID_pt _+ β_3_*Disease_pt_*PaperID_pt _+ β_4_*TestID_pt _+ β7*Disease_pt_*TestID_pt _+ β_6_*TestID_pt_*PaperID_pt _+ β_7_*Disease_pt_*TestID_pt_*PaperID_pt_

implements the decomposition of the performance. Model (2) is fitted to the (repeated measures) grouped binary data, where the 2-by-2 tables of gold-standard versus test results are extracted from each published paper. In the model (2) Result is an integer-valued variable for positive test result (depending on software choice, for grouped binary data, usually Result is replaced by number of positive test results over the total sample size, for each group); Disease is an indicator for actual presence of disease, ascertained by the gold standard; PaperID is a categorical variable for papers included in the meta-analysis; and TestID is a categorical variable for tests included. Regression coefficients ***β_2 _***to ***β_7 _***can be vector valued, meaning having several components, so the corresponding categorical variables should be represented by suitable number of indicator variables in the model. Indexes p and t signify paper p and test t. They define the repeated measures structure of the data [10]. Note model (2) fits the general case where there are two or more tests available for the disease, where each test has been studied in one or more papers. Some of the papers may have studied more than one test; hence the results are not independent. Also the collection of tests studied may change from one paper to the other, hence incomplete matched groups.

From model (2) one can show that

*LOR_pt _*= *β_1 _*+ β_3_*PaperID_pt _+ β_5_* *TestID_pt _*+ β_7_*TestID_pt_*PaperID_pt_

and therefore the difference between performance of two tests i and j, measured by LOR, is

*LOR_pi _*- *LOR_pj _*= β_5_* *TestID_pi _*- β_5_* *TestID_pj _*+ β_7_*TestID_pi_*PaperID_pi _- β_7_*TestID_pj_*PaperID_pj_

where we identify *δ*_i,j _of the decomposition model (1) with the β_5_* *TestID_pi _*- β_5_*TestID_pj_, and identify *δ*_i,j,p _with β_7_*TestID_pi_*PaperID_pi _- β_7_*TestID_pj_*PaperID_pj_.

If there is an obvious and generally accepted diagnostic test that can serve as a reference category (RefCat) to which other tests can be compared, then a "simple" parameterization for tests is sufficient, However, usually it is not the case. When there is no perceived referent test to which the other tests are to be compared, a "deviation from means" coding is preferred for the tests. Using the deviation parameterization for both TestID and PaperID in the model (2), one can show that β_5_*TestID_pt _is the average deviation of the LOR of test t from the overall LOR (the *β_1_*), where the overall LOR is the average over all tests and all papers. Therefore β_5_*TestID_pt _of model (2) will be equivalent to the *δ*_i,j _of the decomposition model (1), and β_7_*TestID_pt_*PaperID_pt _equivalent to *δ*_i,j,p_.

### Proportional odds ratio model

Model (2) expands each study to its original sample size, and uses patients as primary analysis units. Compared to a random-effects model where papers are the primary analysis units, it has more degrees of freedom. However, in a real case, not every test is studied in every paper. Rather majority of tests are not studied in each paper. Therefore the data structure of tests-by-papers is incomplete with many unmeasured cells. The three-way interaction model (2) may become over-parameterized. One may want to drop the term β_6_*Disease_pt_*TestID_pt_*PaperID_pt_. Then for the reduced model

*(3) logit(Result_pt_) *= *β_0 _*+ β_1_*Disease_pt _+ β_2_*PaperID_pt _+ β_3_*Disease_pt_*PaperID_pt _+ β_4_*TestID_pt _+ β_5_*Disease_pt_*TestID_pt_

we have *LOR_pt _*= *β_1 _*+ β_3_*PaperID_pt _+ β_5_* *TestID_pt_*, where the paper and test effects are completely separate. We call this reduced model the Proportional Odds Ratio (POR) model, where the ratio of odds ratios of two tests is assumed to be constant across papers, while odds ratio of each test is allowed to vary across the papers. Note the difference with the proportional odds model where ratio of odds is assumed to be constant [14]. In the POR model

*(4) OR_pt _*= OR_p _*  , *t *= *1*, *2*, ..., *k*, *p *= *1*, *2*, ..., *m*

where t is an index for the *k *diagnostic tests, and p is an index representing the m papers included in the analysis. *OR_p _*is a function capturing the way OR changes across papers. Then to compare two diagnostic tests i and j

*OR_pi _*/ *OR_pj _*= 

where the ratio of the two ORs depends only on the difference between the effect estimates of the two tests, and is independent of the underlying *OR_p _*across the papers. Thus the model makes no assumptions about the shape of *OR_p _*(and in particular homogeneity of ORs) but merely specifies a relationship between the ORs of the two tests.

One may want to replace the PaperID variable with a smooth function of FPR or TPR, such as natural restricted cubic splines. There are two potential advantages. This may preserve some degrees of freedom, where one can spend by adding covariates to the model to measure their potential effects on the performance of the diagnostic tests. Thus one would be able to explain why performance of the same test varies across papers. Also, this allows plotting a ROC curve where the OR is not constant across the curve, a flexible ROC (HetROC) curve.

*(5) logit(Result_pt_) *= *β_0 _*+ β_1_*Disease_pt _+ β_2_*S(FPR_pt_) + β_3_*Disease_pt_*S(FPR_pt_) + β_4_*TestID_pt _+ β_5_*Disease_pt_*TestID_pt _+ β_6_*X_pt _+ β_5_*Disease_pt_*X_pt_

To test the POR assumption one may use model (2) where the three-way interaction of Disease and TestID with PaperID is included. However, in majority of real datasets this would mean an over-parameterized model. Graphics can be used for a qualitative checking of the POR assumption. For instance, the y-axis can be LOR, while the x-axis is paper number. To produce such plot, it may be better to have the papers ordered in some sense. One choice is to compute an unweighted average of (observed) ORs of all the tests the paper studied, and use it as the OR of that paper. Then sort the papers based on such ORs. The OR of a test may vary from one paper to the other (with no restriction), but the POR assumption is that the ratio of ORs of two tests remains the same from one paper to another. If one shows ORs of a test across papers by a smooth curve, then one expects that the two curves of the two tests are proportional to each other. In the log-OR scale, this means the vertical distance of the two curves remains the same across the x-axis. To compute the observed LOR for a test in a paper one may need to add some value (like 1/2) to the cell counts, if some cell counts are zero. However, this could introduce some bias to the estimates.

Among the approaches for modeling repeated-measures data, we use generalized estimating equations to estimate the marginal logistic regression [15]. Software is widely available for estimation of parameters of a marginal POR model. These include SAS (genmod procedure), R (function geese), and STATA (command xtgee), with R being freely available open source software [16].

One may use a non-linear mixed effects modeling approach on the cell-count data for estimation of parameters of the POR model. The Paper effect is declared as random, and interaction of the random effect with Disease is included in the model, as indicated in model (2). However, such mixed effects non-linear models are hard to converge, especially for datasets where there are many papers studying only one or a small number of the included tests (such as the dataset presented as example in this paper). If the convergence is good, it may be possible to fit a mixed model with the interaction of Disease, Test, and the Paper random effect. Such model relaxes the POR assumption, besides relaxing the assumption of OR-homogeneity. In other words, one can use the model to quantitatively test the POR assumption. One should understand that the interpretation of LOR estimate from a marginal model is of a population-average, while that of a mixed model is a conditional-average. Therefore there is a slight difference in their meaning.

### Expanding the proportional odds ratio model

One may use the frameworks of the generalized linear models (GLM) and the generalized estimating equations (GEE) to extend the POR model and apply it to different scenarios. By using suitable GLM link function and random component [[17], p.72], one may fit the POR model to multi-category diagnostic tests, like baseline-category logits, cumulative logits, adjacent-categories and continuation-ratio logits [[17], chapter 8]. A loglinear 'Proportional Performance' (PP) regression may be fitted to the cell counts, treating them as Poisson. Also, one may fit the PP model to the LORs directly, assuming a Gaussian random component with an identity link function. Comparing GEE estimates by fitting the model to 2-by-2 tables versus GEE estimates of the model fitted directly on LOR versus a Mixed model fitted on LOR, usually statistical power decreases across the three. Also, there is issue of incorporation of sample sizes that differ across studies. Note some nuisance parameters, like coefficients of all main effects and the intercept, won't need to be estimated as they are no longer present in the model fitted directly on LORs.

One may avoid dichotomizing results of the diagnostic test by using the 'likelihood ratio' as the performance measure, and fitting a PP model to such continuous outcome. For a scenario where performance of a single test has been measured multiple times within the same study, for example with different diagnostic calibrations (multiple thresholds), the POR estimated by the GEE incorporates data dependencies. When there is a multi-layer and/or nested clustering of repeated measures, software to fit a mixed-effects POR model may be more available than an equivalent GEE POR.

When POR is implemented by a logistic regression on 2-by-2 tables, it uses a grouped binary data structure. It takes a minimal effort to fit the same logistic model to the "ungrouped" binary data, the so-called "individual level" data.

Methods of meta-analysis that allow for different outcomes (and different numbers of outcomes) to be measured per study, such as that of Gleser and Olkin [18], or DuMouchel [19], may be used to implement the POR model. This would prevent conducting parallel meta-analyses that is usually less efficient.

## Results

### Deep vein thrombosis

To demonstrate how to fit the POR model, we use a recent meta-analysis of diagnostic tests for deep vein thrombosis (DVT) by Heim et al. [20]. In this meta-analysis there are 23 papers and 21 tests, comprising 483 potential performance measurements, while only 66 are actually observed, thus 86% of cells are not measured. We fitted the reduced marginal logistic regression model (3). Table 1 shows the parameter estimates for Test effects. SAS code to estimate the parameters is provided [see additional file 1].Data files are provided in Additional file 2.

**Table 1:** Parameter estimates for test effects

**Coefficient**		**Test**	Deviation*	**95% Confidence Limits**	p value**
***β_5_***†	1	Asserachrom	0.524	0.2293, 0.8186	0.0005
	2	Auto Dimertest	0.222	-0.1466, 0.5912	0.2376
	3	BC D-Dimer	-0.993	-2.4195, 0.4333	0.1724
	4	D-Dimer test	0.225	0.1, 0.3494	0.0004
	5	Dimertest	-2.092	-2.3392, -1.8439	<.0001
	6	Dimertest EIA	-0.929	-1.1756, -0.6825	<.0001
	7	Dimertest GOLD EIA	-0.193	-0.4784, 0.0935	0.1871
	8	Dimertest II	-0.731	-0.9774, -0.4843	<.0001
	9	Enzygnost	0.399	0.1209, 0.6766	0.0049
	10	Fibrinostika	0.857	0.6865, 1.0266	<.0001
	11	IL Test	0.809	0.0914, 1.5256	0.0271
	12	Instant I.A.	0.558	0.216, 0.9006	0.0014
	13	Liatest	-0.143	-0.3375, 0.0511	0.1486
	14	LPIA	0.182	-0.0354, 0.3997	0.1007
	15	Minutex	-0.323	-0.8394, 0.193	0.2197
	16	Nephelotex	0.654	0.4325, 0.8745	<.0001
	17	NycoCard	-0.797	-1.0434, -0.5506	<.0001
	18	SimpliRED	0.393	0.1467, 0.6398	0.0018
	19	Tinaquant	0.703	0.0113, 1.3948	0.0464
	20	Turbiquant	-0.328	-1.6596, 1.0032	0.629
	21	VIDAS	1.004	0.365, 1.6424	0.0021
*β_1_*		Overall LOR	2.489	2.4175, 2.5606	< .0001***

* estimate of deviation from overall LOR

** p-value for null hypothesis of Deviation = 0

***p-value for null hypothesis of LOR = 0

*† LOR(Result_pt_) *= *β_1 _*+ β_3_*PaperID_pt _+ β_5_*TestID_pt_

Since we have used deviation contrast for the variables, estimate of *β_1 _*is the "overall mean" for the log-OR. This is similar to an ANOVA analysis where the overall mean is estimated by the model. Therefore the average OR is equal to exp(2.489) = 12.049. Components of ***β_5 _***estimate deviation of LOR of each test from the overall LOR. Software gives estimates of SEs, plus confidence intervals and p-values, so inference is straightforward.

**Figure 1:**
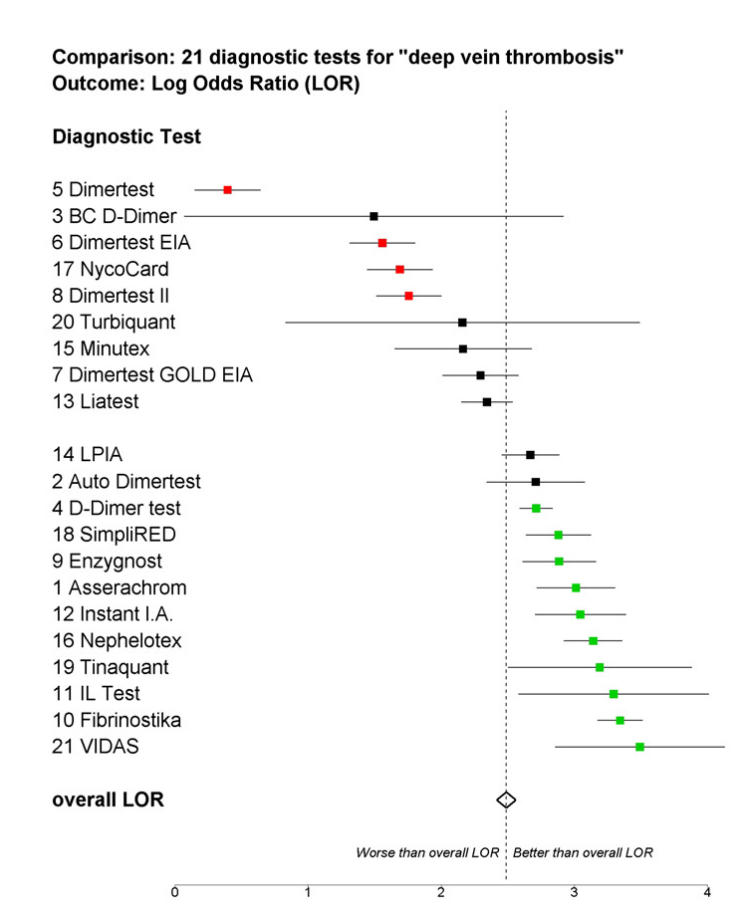
Comparing performance of each diagnostic test to the overall LOR

A forest plot may be used to present the results of the modeling in a graphical way. This may connect better with clinically oriented audience. In Figure 1 we have sorted the 21 tests based on their LOR estimate.

The horizontal axis is log-OR, representing test performance. The dashed vertical line shows overall mean LOR. For each diagnostic test the solid square shows the LOR, while the horizontal line shows the corresponding 95% CI. If the horizontal line does not intersect the vertical line, the test is significantly different from the overall mean LOR.

Note that the CIs in the plot are computed by adding the overall LOR to the CI for the deviation effect of each particular test. This ignores the variability of the overall LOR estimate. One can estimate the LOR of a test and its CI more accurately by some extra computations, or by fitting a slightly modified model. A method is illustrated and implemented [see additional file 1]. However, the gain in accuracy was small in this particular example. The model also estimates paper effects. However, one may not be interested in those primarily.

One can translate LOR to other measures of test performance. There are numerous types of these measures. We provide code to convert the LOR estimated by the POR model to such measures. Note that majority of them, unlike LOR, are in pairs. This means in order to compare two tests, one needs to use two numbers to represent each single test. For example, sensitivity-specificity is a pair. If a test has a higher sensitivity than the other test, while having a lower specificity, it is not immediately clear which test is better. Also, note that some performance measures are independent of disease prevalence, while others depend on prevalence. This means the same test would perform differently for populations with different disease prevalence.

Note in order to compute some of the performance measures, one needs to assume a prevalence and sensitivity or specificity. We assumed a disease prevalence of 40%, and a specificity of 90%, for Table 2, as the tests are mainly used for ruling out the DVT.

**Table 2:** Other performance measures for the 21 diagnostic tests of DVT

	**Diagnostic Test**	**DOR**	**Prev.**	**Spec.**	**Sens.**	**AUC**	**PPV**	**NPV**	**LRAT**	**LRNT**	**PTO**	**PTOAT**	**PTONT**	**PTPAT**	**PTPNT**
1	Asserachrom	20.3	0.4	0.9	0.693	0.888	0.822	0.815	6.933	0.341	0.667	4.622	0.227	0.822	0.185
2	Auto Dimertest	15.0	0.4	0.9	0.626	0.864	0.807	0.783	6.258	0.416	0.667	4.172	0.277	0.807	0.217
3	BC D-Dimer	4.5	0.4	0.9	0.332	0.732	0.688	0.669	3.315	0.743	0.667	2.210	0.495	0.688	0.331
4	D-Dimer test	15.1	0.4	0.9	0.626	0.865	0.807	0.783	6.263	0.415	0.667	4.175	0.277	0.807	0.217
5	Dimertest	1.5	0.4	0.9	0.142	0.566	0.486	0.611	1.419	0.953	0.667	0.946	0.636	0.486	0.389
6	Dimertest EIA	4.8	0.4	0.9	0.346	0.741	0.697	0.674	3.459	0.727	0.667	2.306	0.485	0.697	0.326
7	Dimertest GOLD EIA	9.9	0.4	0.9	0.525	0.826	0.778	0.740	5.248	0.528	0.667	3.499	0.352	0.778	0.260
8	Dimertest II	5.8	0.4	0.9	0.392	0.766	0.723	0.689	3.920	0.676	0.667	2.613	0.450	0.723	0.311
9	Enzygnost	18.0	0.4	0.9	0.666	0.879	0.816	0.802	6.661	0.371	0.667	4.440	0.247	0.816	0.198
10	Fibrinostika	28.4	0.4	0.9	0.759	0.910	0.835	0.849	7.592	0.268	0.667	5.061	0.178	0.835	0.151
11	IL Test	27.0	0.4	0.9	0.750	0.907	0.833	0.844	7.503	0.277	0.667	5.002	0.185	0.833	0.156
12	Instant I.A.	21.1	0.4	0.9	0.701	0.890	0.824	0.818	7.006	0.333	0.667	4.671	0.222	0.824	0.182
13	Liatest	10.4	0.4	0.9	0.537	0.831	0.782	0.745	5.371	0.514	0.667	3.581	0.343	0.782	0.255
14	LPIA	14.5	0.4	0.9	0.616	0.861	0.804	0.779	6.163	0.426	0.667	4.109	0.284	0.804	0.221
15	Minutex	8.7	0.4	0.9	0.492	0.813	0.766	0.727	4.921	0.564	0.667	3.281	0.376	0.766	0.273
16	Nephelotex	23.2	0.4	0.9	0.720	0.897	0.828	0.828	7.202	0.311	0.667	4.801	0.207	0.828	0.172
17	NycoCard	5.4	0.4	0.9	0.376	0.758	0.715	0.684	3.763	0.693	0.667	2.509	0.462	0.715	0.316
18	SimpliRED	17.9	0.4	0.9	0.665	0.878	0.816	0.801	6.648	0.372	0.667	4.432	0.248	0.816	0.199
19	Tinaquant	24.3	0.4	0.9	0.730	0.900	0.830	0.833	7.300	0.300	0.667	4.867	0.200	0.830	0.167
20	Turbiquant	8.7	0.4	0.9	0.491	0.812	0.766	0.726	4.909	0.566	0.667	3.273	0.377	0.766	0.274
21	VIDAS	32.9	0.4	0.9	0.785	0.918	0.840	0.863	7.851	0.239	0.667	5.234	0.159	0.840	0.137

DOR = Diagnostic Odds Ratio

Prev. = Prevalence

Spec. = Specificity

Sens. = Sensitivity

AUC = Area Under Curve (assuming homogeneous OR)

PPV = Positive Predictive Value

NPV = Negative Predictive Value

LRAT = Likelihood Ratio For Abnormal Test

LRNT = Likelihood Ratio For Normal Test

PTO = Pre Test Odds

PTOAT = Post Test Odds Of Abnormal Test

PTONT = Post Test Odds Of Normal Test

PTPAT = Post Test Probability Of Abnormal Test

PTPNT = Post Test Probability Of Normal Test

**Figure 2:**
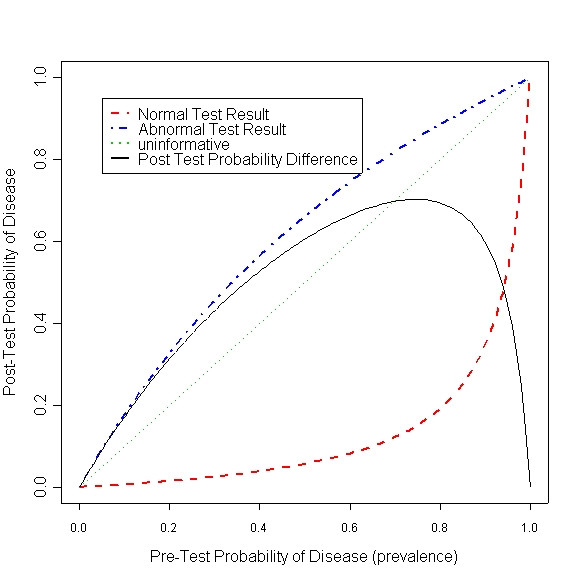
Post-test probability difference for diagnostic test VIDAS

We suggest graphs to compare tests when using such "prevalence-dependent paired performance measures" [21]. In Figure 2 we have used a pair of measures, 'probability of disease given a normal test result' and 'probability of disease given an abnormal test result', the dashed red curve and the dot-and-dash blue curve respectively.

The way one may read the graph is that, given a particular population with a known prevalence of disease like 40%, we perform the diagnostic test on a person picked randomly from the population. If the test turns normal, the probability the person has disease decreases from the average 40% to about 4% (draw a vertical line from point 0.4 on x-axis to the dashed red curve, then draw a horizontal line from the curve to the y-axis). If the test turns abnormal, the probability the person is diseased increases from 40% to about 57%. The dotted green diagonal line represents a test no better than flipping a coin, an uninformative test. The farther the two curves from the diagonal line, the more informative the test is. In other words, the test performs better.

One can summarize the two curves of a test in a single curve, by computing the vertical distance between the two. The solid black curve in the figure is such "difference" curve. It seems this particular test is performing the best in populations with disease prevalence of around 75%.

**Figure 3:**
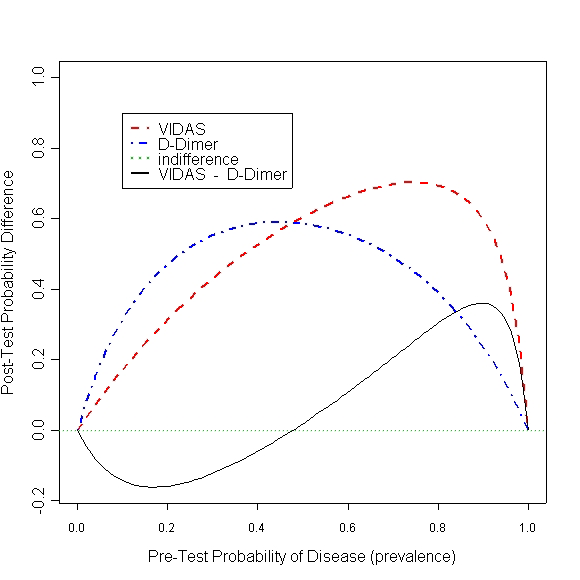
Comparing post-test probability difference for VIDAS – D-Dimer

One can use the difference curve to compare several tests, and study effect of prevalence on the way the tests compare to each other. In Figure 3 two tests VIDAS and D-Dimer from the DVT example are compared. From the model estimates we know that both tests perform better than average. And that VIDAS performs better than D-Dimer.

The black solid curve is comparing the two tests. For populations with low disease prevalence (around 17%), the D-Dimer is performing better than VIDAS. However, when the prevalence is higher (around 90%), VIDAS is preferred. Simultaneous confidence bands around the comparison curve would make formal inference possible.

### Random effects

A nonlinear mixed effects POR model fitted to cell counts of the DVT dataset does not converge satisfactorily. We fitted the mixed model to a subset of the data where only two tests and seven papers are included, Table 3. For codes refer to the additional file 1.

**Table 3:** Data structure for two diagnostic tests

		**Test**	
	**Paper**	Instant I.A.	NycoCard
3	Elias, A. 1996 (171)	X	X
8	Legnani, C. 1997 (81)	X	X
11	Leroyer, C. 1997 (448)	X	
12	Scarano, L. 1997 (126)	X	X
13	van der Graaf, F. 2000 (99)	X	X
21	Wijns, W. 1998 (74)	X	
22	Kharia, HS. 1998 (79)		X
	TOTAL	6	5

Five of the seven papers have studied both the tests. Result of SAS Proc NLMixed still is sensitive to initial values of parameters. The three-way interaction term of disease, test, and paper in the mixed model (where POR is not assumed) is insignificant, Table 4. A POR assumption for the two tests may be acceptable.

**Table 4:** Comparing parameter estimates from three models

	POR-relaxed Mixed *	**POR Mixed**	**POR Marginal**
**overall LOR**	1.389 (0.993, 1.786)	0.868 (0.568, 1.169)	2.593 (2.522, 2.664)
**Test (NycoCard)**	-0.903 (-1.811, 0.006)	-0.93 (-1.104, -0.755)	-0.561 (-0.829, -0.293)
Test*Paper	0.016 (-1.556, 1.588)	---	---

* *logit(Result) *= *β_0 _*+ β_1_*Disease + β_2_*PaperID + β_3_*Disease*PaperID + β_4_*TestID + β_5_*Disease*TestID + β_6_*Disease*TestID*PaperID

The estimate of overall LOR from both the POR-mixed model and POR-marginal model are significantly different from zero. However, the mixed model estimate of LOR is much smaller than the marginal one. For non-linear models, the marginal model describes the population parameter, while the mixed model describes an individual's [[15], p.135]. The estimate of deviation of test (NycoCard) from the overall LOR is closer in the two models. Plus the marginal estimate is closer to 0 than the mixed estimate. One expects coefficient estimates of mixed model being closer to zero, compared to the fixed model, while the mixed model CI's being wider.

### Meta-analysis of a single test: the baseline *OR_p _*function

**Figure 4:**
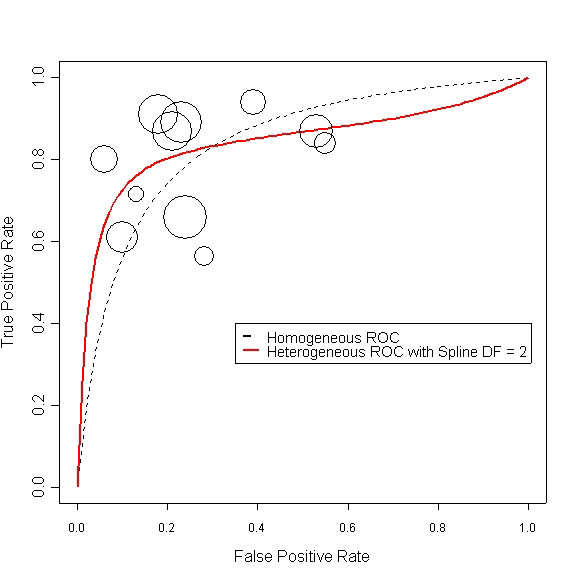
Heterogeneous ROC curve for diagnostic test SimpliRED

Sometimes one may be interested in constructing the ROC curve for the diagnostic test. A homogeneous ROC curve assumes the performance of the test (as measured by LOR) is the same across the whole range of specificity. However, this assumption may be relaxed in a HetROC. We fitted a simplified version of model (5) for test SimpliRED,

*logit(Result_pt_) *= *β_0 _*+ β_1_*Disease_pt _+ β_2_*S(FPR_pt_) + β_3_*Disease_pt_*S(FPR_pt_)

where index t is fixed, and then used estimates of the coefficients to plot the corresponding HetROC, Figure 4.

The eleven papers that studied test SimpliRED are shown by circles where the area is proportional to the sample size of the study. The black dashed curve is ROC curve assuming homogeneous-OR. The red solid curve relaxes the assumption, hence a heterogeneous ROC curve. The amount of smoothing of the curve can be controlled by the "degree-of-freedom" DF parameter. Here we have used a DF of 2. Codes to make such plots are presented in the additional file 1.

### Model checking

**Figure 5:**
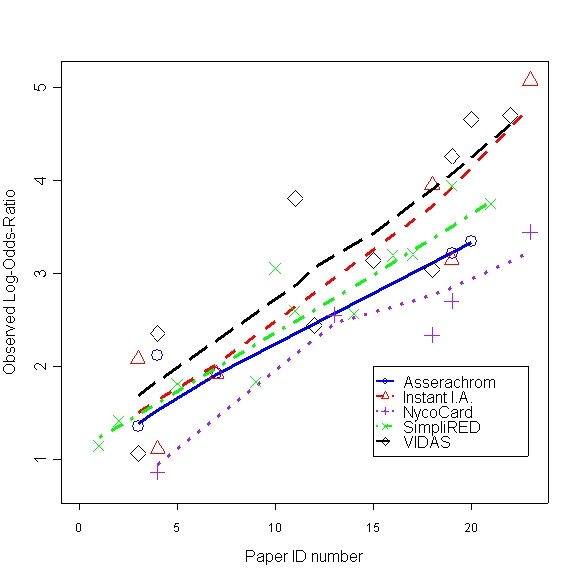
Observed log-odds-ratios of each diagnostic test

Checking the POR assumption, model (2) may be used to reject significance of the three-way interaction term. However, the dataset gathered for the DVT meta-analysis is such that no single paper covers all the tests. Moreover, out of 21, there are 7 tests that have been studied in only one paper. For Figure 5 we chose tests that have been studied in at least 5 of the 23 papers. There are 5 such tests. Note that even for such "popular" tests, out of 10 pairwise comparisons, 3 are based on only one paper (so no way to test POR). Four comparisons are based on 4 papers, one based on 3 papers, and the remaining two comparisons are based on 2 papers.

We sorted the papers, the x-axis, based on average LOR within that paper. We fitted Lowess smooth lines to the observed LORs of each test separately. Figure 5 shows the smooth curves are relatively parallel. Note the range of LORs of a single test. The LORs vary considerably from one paper to the other. Indeed the homogeneity-of-ORs assumption is violated in four of the five tests.

Also, to verify how good the model fits the data, one may use an observed-versus-fitted plot. Plots or lists of standardized residuals may be helpful finding papers or tests that are not fitted well. This may provide a starting point for further investigation.

## Discussion

A comparison of the relative accuracy of several diagnostic tests should ideally be based on applying all the tests to each of the patients or randomly assigning tests to patients in each primary study. Obtaining diagnostic accuracy information for different tests from different primary studies is a weak design [3]. Comparison of the accuracy of two or more tests within each primary study is more valid than comparison of the accuracy of two or more tests between primary studies [22]. Although a head-to-head comparison of diagnostic tests provides more valid results, there are real-world practical questions that meta-analysis provides an answer that is more timely and efficient than a single big study [23]. Meta-analysis can potentially provide better understanding by examining the variability in estimates, hence the validity versus generalizability (applicability). Also, there may be tests that have never been studied simultaneously in a single study, hence meta-analysis can "reconstruct" such a study of diagnostic tests.

### Relaxing the assumption of OR homogeneity

In meta-analysis of two (or more) diagnostic tests, where attention is mainly on the difference between performances of two tests, having a homogeneous estimate of performance of each single test is of secondary importance, and it may be treated as nuisance. The POR model assumes differences between LORs of two tests are the same across all papers, but does not assume the OR of a test is the same in every paper. Hence no need for homogeneity of OR of a test across papers that reported it, but shifting the assumption one level higher to POR.

### Common versus average effect size

The POR model uses "deviation from means" parameterization. Then one does not need to drop the interactions coefficient ***β_3 _***in the model *logit(Result) *= *β_0 _*+ β_1_*Disease + β_2_*PaperID + β_3_*Disease*PaperID, to interpret *β_1_*, the overall LOR. This means the POR model explicitly accepts that performance of the diagnostic test varies across the papers, but at the same time estimates its mean value. McClish explains if a test for OR homogeneity shows heterogeneity, there may be no 'common' measure to report, but still there is an 'average' measure one can report. [13]

### Advantages of using 2-by-2 tables

We demonstrated how to fit the POR model to the cell counts, rather than to the OR values. This, we believe, has several advantages. 1. One does not need assuming normality of some summary measure. This results in binomial distributional assumption that is more realistic. 2. Also, different study sample sizes are incorporated into the POR model without faulty bias-introducing weighting schemes, as shown by Mosteller & Chalmers [25]. And extension of the POR model to individual level patient data is much easier. 3. The effective sample size for a meta-analysis by a random model is the number of papers included, which is usually quite small. There is a great danger for overfitting. And the number of explanatory variables one could include in the model is very restricted. Since we use the grouped binary data structure, the patients are the effective sample size, hence much bigger degrees of freedom.

The way the random-effects model is usually implemented is by extracting OR from each paper, and assuming LOR being normally distributed. Then the distinction between the two types of mistakes (FNR and FPR, or equivalently TPR and FPR) is lost, since one enters the LOR as datapoints into the model. The bivariate model by Houwelingen et al [26] tries to fix this, by entering two datapoints into the model for each test from each paper. A fourth advantage of fitting the POR model to the cell counts is that the two types of mistakes are included in the model. Consider the logistic regression *logit(Result) *= *β_0 _*+ β_1_*Disease + β_2_*PaperID . Then we have log(true positive/false negative) = *β_0 _*+ *β_1 _*+ β_2_*PaperID. Substituting a value for the covariate (here PaperID) such as a modal or average value, and using the model estimates for the betas, one gets the log-odds. Then one exponentiates it to get the TP/FN, call it Q. Now it is easy to verify that sensitivity = Q/(1+Q). Likewise we have log(false positive/true negative) = *β_0 _*+ β_2_*PaperID, that we call = log(W). Then specificity = 1/(1+W). Also, one can apply separate weights to the log(true positive/false negative) and log(false positive/true negative), to balance the true positive and false positive rates for decision making in a particular clinical practice.

When collecting papers from biomedical literature for meta-analysis of a few diagnostic tests, it is hard to come up with a complete square dataset, where every paper has included all the tests of interest. Usually the dataset contains missing values, and a case-wise deletion of papers with missing tests means a lot of data is thrown away. A method of analysis that can utilize incomplete matched groups may be helpful. The POR model allows complex missing patterns in data structure. Convergence of marginal POR model seems much better than non-linear mixed model, when fitted to cell counts of incomplete matched groups. This is an advantage for using GEE to estimate POR.

The fact that one can use popular free or commercial software to fit the proposed models, facilitates incorporation of the POR modeling in the practice of meta-analysis.

### Unwanted heterogeneity versus valuable variability

The POR model utilizes the variation in the observed performance of a test across papers. Explaining when and how the performance of the test changes, and finding the influential factors, is an important step in advancing science. In other words, rather than calling it 'heterogeneity', treated as 'unwanted' and unfortunate, one calls it 'variability' and utilizes the observed variability to estimate and explain when and how to use the agent or the test in order to optimize their effects.

Victor [32] emphasizes that results of a meta-analysis can only be interpreted if existing heterogeneities can be adequately explained by methodological heterogeneities. The POR model estimates effect of potential predictors on between-study variation, hence trying to 'explain' why such variation exists.

The POR model incorporates risk of events in the control group via a predictor, such as observed prevalence, hence a 'control rate regression'. [26]

### ROC curve

Although implementing the HetROC means that one accepts the diagnostic test performs differently in different FPRs along the ROC curve, in some implementations of HetROC, such as method of summary ROC, one compares tests by a single point of their respective ROCs. This is not optimal. (The Q test of the SROC method is a single point test, where that point on the ROC may not be the point for a specific cost-benefit case.) In such method although one produces a complete SROC, but one does not use it in comparing the diagnostic tests. In the POR model, one uses LOR as the measure for diagnostic discrimination accuracy, and builds statistical test based on the LOR-ratio, hence the test corresponds to whole ROCs (of general form).

The ROC graph was designed in the context of the theory of signal detectability [27,28]. ROC can be generated in two ways, by assuming probability distribution functions (PDFs) for the two populations of 'diseased' and 'healthy', or by algebraic formulas [29]. Nelson claims the (algebraic) ROC framework is more general than the signal detection theory (and its PDF-based ROC) [5]. The location-scale regression models implement ROC via PDFs, while the method of summary-ROC uses algebraic approach. The POR model uses a hybrid approach. While POR may be implemented by logistic regression, the smoothing covariate resembles the algebraic method. Unlike location-scale regression models that use two equations, POR uses one equation, hence it is easier to fit by usual statistical packages. One may use a five-parameter logistic to implement the HetROC. However, the model cannot be linearized, then according to McCullagh [14] it won't have good statistical properties. The POR model not only relaxes assumption of Var1/Var2 = 1, where Var1 and Var2 are variances of the two underlying distributions for the two populations, but even monotonicity of ROC. Hence the model can be used to represent both asymmetric ROCs and non-regular ROCs (singular detection).

In building HetROC curve, the POR model accommodates more general heterogeneous ROCs than SROC, because it uses nonparametric smoother instead of arbitrary parametric functions used in SROC method. When in the POR model the smoother covariate is replaced by log{TPR*FPR/ [(1-TPR)*(1-FPR)]}, a HetROC similar to SROC of Moses et al is produced.

When one uses a smooth function of FPR in the POR model, it is equivalent to using a function of outcome as predictor. This resembles a 'transition model'. Ogilvie and Creelman [30] claim that for estimating parameters of a best fitting curve going through observed points in the ROC space, least squares is not good since both axes are dependent variables and subject to error. They claim maximum likelihood is a preferred method of estimation. Crouchley and Davies [31] warn that, although GEE is fairly robust, it becomes inconsistent if any of the covariates are endogenous, like a previous or related outcome or baseline outcome. They claim a mixed model is better for studying microlevel dynamics. We have observed that the smooth HetROC curve may become decreasing at right end, due to some outlier points. Using less smoothing in the splines may be a solution.

When there is only one diagnostic test, and one is mainly interested in pooling several studies of the same test, the POR model estimates effect sizes that are more generalizable. By using the smoother (instead of PaperID), one fits a sub-saturated model that allows inclusion of other covariates, hence it is possible to estimate effect of study level factors on performance and explain the heterogeneity. Also it does not assume any a priori shape of the ROC, including monotonicity. Plus, it enables graphing of the HetROC. It does not need omission of interaction terms to estimate the overall performance, and it does not need assumption of OR homogeneity. If several performance measurements of the same test is done in a single study, like evaluating the same test with different diagnostic calibrations, the POR model provides more accurate estimates, by incorporating the dependence structure of the data.

### Random effects

When there is heterogeneity between a few studies for the same diagnostic test, one solution to absorb the extra between-study variation is to use a random/mixed effects model. However, Greenland [33] cautions when working with random effect models: 1. if adding random effect changes the inference substantially, it may indicate large heterogeneity, needing to be explained; 2. specific distributional forms for random effects have no empiric, epidemiologic, or biologic justification. So check its assumptions; 3. the summary statistic from random-effect model has no population-specific interpretation. It represents the mean of a distribution that generates effects. Random models estimate unit specific coefficients while marginal models estimate population averages. The choice between unit-specific versus population-average estimates will depend on the specific research questions that are of interest. If one were primarily interested in how a change in a covariate affect a particular individual cluster's mean, one would use the unit-specific model. If one were interested in how change in covariate can be expected to affect the overall population mean, one would use the population-average model. The difference between "unit-specific" models and "population-average" models arises only in the case of a nonlinear link function. In essence random-effect model exchanges questionable homogeneity assumption for a fictitious random distribution of effects. Advantage of a random model is that SE and CI reflect unaccounted-for sources of variation, and its drawback is that simplicity of interpretation is lost. When residual heterogeneity is small, fixed and random should give same conclusions. Inference about the fixed effects (in a mixed model) would apply to an entire population of cases defined by random effect, while the same coefficient from a fixed model apply only to particular units in the data set. Crouchley and Davies [31] explain one of the drawbacks of their random model is that it rapidly becomes over-parameterized, and also may encounter multiple optima.

### Follow-ups

We suggest these follow-ups: 1. the POR model has been implemented both by marginal and mixed models. It would be useful to implement a marginalized mixed POR model; 2. in clinical practice, usually a group of diagnostic tests is performed on an individual, for a particular disease. Some of these tests are requested simultaneously and some in sequence. It would be useful, and practically important, to extend the POR model such that it incorporates such sequence of testing and a priori results; 3. the utility of POR model may be extended to meta-analysis of therapeutics.

## Competing interests

The author(s) declare that they have no competing interests.

## Authors' contributions

MSS conceived of the model, and participated in its design and implementation. JS participated in implementation of the model and performing of the example analysis. Both authors read and approved the final manuscript.

## Pre-publication history

The pre-publication history for this paper can be accessed here:



## Supplementary Material

Additional File 1 In this file we present sample codes for a few of the models presented in the paper. The estimation mostly has been done in SAS, while the graphing (and some model-fitting) has been done in R. Click here for file

Additional File 2 This zipped file contains 8 data files, in the .csv (comma separated value) and .xls (MS Excel) formats. They are to be used with the SAS and R codes we presented in the Appendix [additional file 1]. Five files are for the SAS codes presented in the Appendix. The file names are "data5.xls", "data5_t12&17.xls", "u125.xls", "data5_t18.xls", "data6.xls". Three files are for the R codes presented in the Appendix. The file names are "obsVSfit.csv", "dataNewExcerpt2.csv", and "data6_lor2.csv". Click here for file
