# Supplementary material for: Proportional odds ratio model for comparison of diagnostic tests in meta-analysis
Source: BMC Med Res Methodol. 2004 Dec 10;4:27. doi: 10.1186/1471-2288-4-27 (PMC539279; doi:10.1186/1471-2288-4-27)
Supplement: Additional File 1 — In this file we present sample codes for a few of the models presented in the paper. The estimation mostly has been done in SAS, while the graphing (and some model-fitting) has been done in R. [file 1471-2288-4-27-S1.doc]

##### Appendix

In this appendix we present sample codes for a few of the models presented in the paper. The estimation mostly has been done in SAS, while the graphing (and some model-fitting) has been done in R.

**1. SAS codes**

**1.1. SAS code for marginal model with deviation parameterization of categorical variables**

Statistical model:

*logit(Result_pt_) = β_0_ + β_1_*Disease_pt_ +* ***β_2_*****PaperID_pt_ +* ***β_3_*****Disease_pt_*PaperID_pt_ +* ***β_4_*****X_pt_ +* ***β_5_*****Disease*X_pt_ +* ***β_6_*****TestID_pt_ +* ***β_7_*****Disease_pt_*TestID_pt_*

Reading data from an MS Excel file:

**PROC** **IMPORT** OUT= WORK.dvt

DATAFILE= "C:\Documents and Settings\hes\Desktop\data5.xls"

DBMS=EXCEL2000 REPLACE;

GETNAMES=YES;

**RUN**;

Here is a sample of the data file, the first 20 rows:

| **n** | **result** | **disease** | **paper** | **paperLabel** | **gold** | **goldLabel** | **setting** | **settingLabel** | **prvlnc** | **cprvlnc** | **test** | **testLabel** |
| --- | --- | --- | --- | --- | --- | --- | --- | --- | --- | --- | --- | --- |
| 36 | 17 | 0 | 1 | 2Brenner, B. 1995 (86) | 1 | US-V | 2 | Outpatient | 58 | 20 | 6 | Dimertest EIA |
| 50 | 6 | 1 | 1 | 2Brenner, B. 1995 (86) | 1 | US-V | 2 | Outpatient | 58 | 20 | 6 | Dimertest EIA |
| 36 | 24 | 0 | 1 | 2Brenner, B. 1995 (86) | 1 | US-V | 2 | Outpatient | 58 | 20 | 8 | Dimertest II |
| 50 | 10 | 1 | 1 | 2Brenner, B. 1995 (86) | 1 | US-V | 2 | Outpatient | 58 | 20 | 8 | Dimertest II |
| 36 | 22 | 0 | 1 | 2Brenner, B. 1995 (86) | 1 | US-V | 2 | Outpatient | 58 | 20 | 18 | SimpliRED |
| 50 | 3 | 1 | 1 | 2Brenner, B. 1995 (86) | 1 | US-V | 2 | Outpatient | 58 | 20 | 18 | SimpliRED |
| 81 | 36 | 0 | 2 | 4D'Angelo, A. 1996 (103) | 1 | US-V | 1 | In-Mix | 21 | -17 | 21 | VIDAS |
| 22 | 1 | 1 | 2 | 4D'Angelo, A. 1996 (103) | 1 | US-V | 1 | In-Mix | 21 | -17 | 21 | VIDAS |
| 96 | 21 | 0 | 3 | 5Elias, A. 1996 (171) | 1 | US-V | 1 | In-Mix | 44 | 6 | 1 | Asserachrom |
| 75 | 2 | 1 | 3 | 5Elias, A. 1996 (171) | 1 | US-V | 1 | In-Mix | 44 | 6 | 1 | Asserachrom |
| 96 | 60 | 0 | 3 | 5Elias, A. 1996 (171) | 1 | US-V | 1 | In-Mix | 44 | 6 | 4 | D-Dimer test |
| 75 | 20 | 1 | 3 | 5Elias, A. 1996 (171) | 1 | US-V | 1 | In-Mix | 44 | 6 | 4 | D-Dimer test |
| 96 | 29 | 0 | 3 | 5Elias, A. 1996 (171) | 1 | US-V | 1 | In-Mix | 44 | 6 | 9 | Enzygnost |
| 75 | 5 | 1 | 3 | 5Elias, A. 1996 (171) | 1 | US-V | 1 | In-Mix | 44 | 6 | 9 | Enzygnost |
| 96 | 36 | 0 | 3 | 5Elias, A. 1996 (171) | 1 | US-V | 1 | In-Mix | 44 | 6 | 10 | Fibrinostika |
| 75 | 5 | 1 | 3 | 5Elias, A. 1996 (171) | 1 | US-V | 1 | In-Mix | 44 | 6 | 10 | Fibrinostika |
| 96 | 18 | 0 | 3 | 5Elias, A. 1996 (171) | 1 | US-V | 1 | In-Mix | 44 | 6 | 12 | Instant I.A. |
| 75 | 5 | 1 | 3 | 5Elias, A. 1996 (171) | 1 | US-V | 1 | In-Mix | 44 | 6 | 12 | Instant I.A. |
| 96 | 36 | 0 | 3 | 5Elias, A. 1996 (171) | 1 | US-V | 1 | In-Mix | 44 | 6 | 17 | NycoCard |
| 75 | 15 | 1 | 3 | 5Elias, A. 1996 (171) | 1 | US-V | 1 | In-Mix | 44 | 6 | 17 | NycoCard |

Using TRANSREG to compute design matrix for deviation contrasts, for main effects, and for interactions.

**proc** **transreg** data=dvt design;

model class( paper gold setting test /deviations zero='1' '1' '1' '1' cprefix = **1** **4** **7** **1**)

identity(prvlnc / tstandard=center name=(cprevalence));

id n result disease prvlnc cprvlnc;

output out=dvtno1;

**run**;

The above Transreg centralizes the prevalence, but it counts each paper to the number of tests studied by that paper.

Fitting the marginal model by GEE (note three covariates are included; however, none are estimable, mostly due to over-parameterization):

**proc** **genmod** data=dvtno1 ;

class paper test disease gold setting;

model result/n = disease

p2 p3 p4 p5 p6 p7 p8 p9 p10 p11 p12 p13 p14 p15 p16 p17 p18 p19 p20 p21 p22 p23

disease*p2 disease*p3 disease*p4 disease*p5 disease*p6 disease*p7 disease*p8 disease*p9 disease*p10 disease*p11 disease*p12 disease*p13 disease*p14 disease*p15 disease*p16 disease*p17 disease*p18 disease*p19 disease*p20 disease*p21 disease*p22 disease*p23

gold2

disease*gold2

setting2

disease*setting2

cprevalence

disease*cprevalence

t2 t3 t4 t5 t6 t7 t8 t9 t10 t11 t12 t13 t14 t15 t16 t17 t18 t19 t20 t21

disease*t2 disease*t3 disease*t4 disease*t5 disease*t6 disease*t7 disease*t8 disease*t9 disease*t10 disease*t11 disease*t12 disease*t13 disease*t14 disease*t15 disease*t16 disease*t17 disease*t18 disease*t19 disease*t20 disease*t21

/ dist=bin link=logit;

repeated subject=paper / within=test*disease type=exch covb corrw;

output out=dvtrsdl resraw=rawr reschi=pearsonr;

**run**;

This is a rerun of the same model but with a different RefCat, so that all the coefficients are estimated by the software.

**proc** **transreg** data=dvt design;

model class( paper gold setting test /deviations zero='2' '1' '1' '2' cprefix = **1** **4** **7** **1**)

identity(prvlnc / tstandard=center name=(cprevalence));

id n result disease prvlnc cprvlnc;

output out=dvtno2;

**run**;

**proc** **genmod** data=dvtno2 ;

class paper test disease gold setting;

model result/n = disease

p1 p3 p4 p5 p6 p7 p8 p9 p10 p11 p12 p13 p14 p15 p16 p17 p18 p19 p20 p21 p22 p23

disease*p1 disease*p3 disease*p4 disease*p5 disease*p6 disease*p7 disease*p8 disease*p9 disease*p10 disease*p11 disease*p12 disease*p13 disease*p14 disease*p15 disease*p16 disease*p17 disease*p18 disease*p19 disease*p20 disease*p21 disease*p22 disease*p23

gold2

disease*gold2

setting2

disease*setting2

cprevalence

disease*cprevalence

t1 t3 t4 t5 t6 t7 t8 t9 t10 t11 t12 t13 t14 t15 t16 t17 t18 t19 t20 t21

disease*t1 disease*t3 disease*t4 disease*t5 disease*t6 disease*t7 disease*t8 disease*t9 disease*t10 disease*t11 disease*t12 disease*t13 disease*t14 disease*t15 disease*t16 disease*t17 disease*t18 disease*t19 disease*t20 disease*t21

/ dist=bin link=logit;

repeated subject=paper / within=test*disease type=exch covb corrw;

output out=dvtrsdl resraw=rawr reschi=pearsonr;

**run**;

**1.2. SAS code for marginal model for more accurate CI for LOR of a particular test**

When one wants to compute CI for LOR of a particular test, rather than using CI of deviation of the test from the overall LOR (estimated by the previous section), one can use the following code.

**proc** **transreg** data=dvt design;

model

class(test / zero='1' cprefix = **1**)

class( paper gold setting /deviations zero='1' '1' '1' cprefix = **1** **4** **7**)

identity(prvlnc / tstandard=center name=(cprevalence));

id n result disease prvlnc cprvlnc;

output out=dvtno1;

**run**;

**proc** **genmod** data=dvtno1 ;

class paper test disease gold setting;

model result/n = disease

t2 t3 t4 t5 t6 t7 t8 t9 t10 t11 t12 t13 t14 t15 t16 t17 t18 t19 t20 t21

disease*t2 disease*t3 disease*t4 disease*t5 disease*t6 disease*t7 disease*t8 disease*t9 disease*t10 disease*t11 disease*t12 disease*t13 disease*t14 disease*t15 disease*t16 disease*t17 disease*t18 disease*t19 disease*t20 disease*t21

p2 p3 p4 p5 p6 p7 p8 p9 p10 p11 p12 p13 p14 p15 p16 p17 p18 p19 p20 p21 p22 p23

disease*p2 disease*p3 disease*p4 disease*p5 disease*p6 disease*p7 disease*p8 disease*p9 disease*p10 disease*p11 disease*p12 disease*p13 disease*p14 disease*p15 disease*p16 disease*p17 disease*p18 disease*p19 disease*p20 disease*p21 disease*p22 disease*p23

gold2

disease*gold2

setting2

disease*setting2

cprevalence

disease*cprevalence

/ dist=bin link=logit;

repeated subject=paper / within=test*disease type=exch covb corrw;

output out=dvtrsdl resraw=rawr reschi=pearsonr;

**run**;

One needs to change line

class(test / zero='1' cprefix = **1**)

such that zero=’’ is the category number one wants to be RefCat. Also needs to change lines

t2 t3 t4 t5 t6 t7 t8 t9 t10 t11 t12 t13 t14 t15 t16 t17 t18 t19 t20 t21

disease*t2 disease*t3 disease*t4 disease*t5 disease*t6 disease*t7

such that the omitted RefCat name matches with these lines in Genmod. Then one reruns the model repeatedly, one for each category. Then beta of Disease would be LOR of that particular test.

**1.3. SAS code for marginal model for equivalence class of a (best) test**

SAS code to compare tests to a good RefCat (a test with good LOR), like VIDAS.

This model is specifically good to answer the question “for disease DVT, what tests are included in the equivalence-class of best diagnostic tests?” However, note the design of the meta-analysis was not of an equivalence-trial.

**proc** **transreg** data=dvt design;

model

class(test / zero='21' cprefix = **1**)

class( paper gold setting /deviations zero='1' '1' '1' cprefix = **1** **4** **7**)

identity(prvlnc / tstandard=center name=(cprevalence));

id n result disease prvlnc cprvlnc;

output out=dvtno1;

**run**;

**proc** **genmod** data=dvtno1 ;

class paper test disease gold setting;

model result/n = disease

t1 t2 t3 t4 t5 t6 t7 t8 t9 t10 t11 t12 t13 t14 t15 t16 t17 t18 t19 t20

disease*t1 disease*t2 disease*t3 disease*t4 disease*t5 disease*t6 disease*t7 disease*t8 disease*t9 disease*t10 disease*t11 disease*t12 disease*t13 disease*t14 disease*t15 disease*t16 disease*t17 disease*t18 disease*t19 disease*t20

p2 p3 p4 p5 p6 p7 p8 p9 p10 p11 p12 p13 p14 p15 p16 p17 p18 p19 p20 p21 p22 p23

disease*p2 disease*p3 disease*p4 disease*p5 disease*p6 disease*p7 disease*p8 disease*p9 disease*p10 disease*p11 disease*p12 disease*p13 disease*p14 disease*p15 disease*p16 disease*p17 disease*p18 disease*p19 disease*p20 disease*p21 disease*p22 disease*p23

gold2

disease*gold2

setting2

disease*setting2

cprevalence

disease*cprevalence

/ dist=bin link=logit;

repeated subject=paper / within=test*disease type=exch covb corrw;

output out=dvtrsdl resraw=rawr reschi=pearsonr;

**run**;

Needs to change line

class(test / zero='1' cprefix = **1**)

such that zero=’’ of the category number one wants to be RefCat. Also needs to change lines

t2 t3 t4 t5 t6 t7 t8 t9 t10 t11 t12 t13 t14 t15 t16 t17 t18 t19 t20 t21

disease*t2 disease*t3 disease*t4 disease*t5 disease*t6 disease*t7

such that the omitted RefCat name matches with these lines in Genmod. Then beta of Disease would be LOR of that particular test (with its CI), and beta of Test are deviation of the rest of tests from RefCat test, and whether significant.

**1.4. SAS code for mixed effects models, marginal model, and their comparison**

Import Excel data

**PROC** **IMPORT** OUT= WORK.dvtt12t17

DATAFILE= "C:\Documents and Settings\hes\Desktop\data5_t12

&17.xls"

DBMS=EXCEL2000 REPLACE;

GETNAMES=YES;

**RUN**;

Put in deviation parameterization:

**proc** **transreg** data=dvtt12t17 design;

model class( paper gold setting test /deviations zero='3' '1' '1' '12' cprefix = **1** **4** **7** **1**)

identity(prvlnc / tstandard=center name=(cprevalence));

id n result disease prvlnc cprvlnc;

output out=dvtno12;

**run**;

Fitting marginal POR model:

**proc** **genmod** data=dvtno12 ;

class paper test disease gold setting;

model result/n = disease

p8 p11 p12 p13 p21 p22

disease*p8 disease*p11 disease*p12 disease*p13 disease*p21 disease*p22

t17

disease*t17

/ dist=bin link=logit;

repeated subject=paper / within=test*disease type=exch covb corrw;

output out=dvtrsdl resraw=rawr reschi=pearsonr;

**run**;

Fit POR (nonlinear) mixed effects model:

**proc** **nlmixed** data=dvtno12 qmax=**1000** noad noadscale;

parms

s2u = **.5**

beta0= -**2.5**

betad= **2.6**

betadp= **.5**

betat17= -**.5**

betadt17= **.5**

;

eta = beta0 + betad*disease + u + betadp*disease*u + betat17*t17 + betadt17*t17*disease

;

expeta=exp(eta);

p = expeta/(**1**-expeta);

model result ~ binomial(n, p);

random u ~ normal(**0**,s2u) subject=paper;

estimate '-betad' -betad;

predict u out=u;

**run**;

**PROC** **EXPORT** DATA= Work.U

OUTFILE= "C:\Documents and Settings\hes\Desktop\u125.xls"

DBMS=EXCEL2000 REPLACE;

**RUN**;

Different initial values may give different convergence, and parameter estimates, but final inference for the highest model term does not change that much.

Fitting a modified POR-relaxed mixed effects model (without the Paper*Test interaction):

**proc** **nlmixed** data=dvtno12 qmax=**1000** noad noadscale;

parms

s2u = **.5**

beta0= -**2.5**

betad= **2.6**

betadp= **.5**

betat17= -**.5**

betadt17= **.5**

betadpt17= **.5**

;

eta = beta0 + betad*disease + u + betadp*disease*u + betat17*t17 + betadt17*t17*disease + betadpt17*t17*disease*u

;

expeta=exp(eta);

p = expeta/(**1**-expeta);

model result ~ binomial(n, p);

random u ~ normal(**0**,s2u) subject=paper;

estimate '-betad' -betad;

predict u out=u;

**run**;

**1.5. SAS code for models for a single test meta-analysis**

Single test case. Choosing SimpliRED.

**PROC** **IMPORT** OUT= WORK.dvtt18

DATAFILE= "C:\Documents and Settings\hes\Desktop\data5_t18.xls"

DBMS=EXCEL2000 REPLACE;

GETNAMES=YES;

**RUN**;

Put in deviation parameterization:

**proc** **transreg** data=dvtt18 design;

model class( paper gold setting /deviations zero='1' '1' '1' cprefix = **1** **4** **7**)

identity(prvlnc / tstandard=center name=(cprevalence));

id n result disease prvlnc cprvlnc;

output out=dvtno1;

**run**;

Marginal (although only one test per paper, but two rows of data):

**proc** **genmod** data=dvtno1 ;

class paper disease gold setting;

model result/n = disease

p5 p6 p7 p10 p13 p14 p15 p17 p18 p19

disease*p5 disease*p6 disease*p7 disease*p10 disease*p13 disease*p14 disease*p15 disease*p17 disease*p18 disease*p19

/ dist=bin link=logit;

output out=dvtrsdl resraw=rawr reschi=pearsonr;

**run**;

NOTE: type=exch gives error. Type=indep gives 0 for all SE of GEE parameters, seemingly due to saturated model. Including covariates, Genmod does not estimate them.

Changing RefCat:

**proc** **transreg** data=dvtt18 design;

model class( paper gold setting /deviations zero='5' '1' '1' cprefix = **1** **4** **7**)

identity(prvlnc / tstandard=center name=(cprevalence));

id n result disease prvlnc cprvlnc;

output out=dvtno5;

**run**;

**proc** **genmod** data=dvtno5 ;

class paper disease gold setting;

model result/n = disease

p1 p6 p7 p10 p13 p14 p15 p17 p18 p19

disease*p1 disease*p6 disease*p7 disease*p10 disease*p13 disease*p14 disease*p15 disease*p17 disease*p18 disease*p19

/ dist=bin link=logit;

output out=dvtrsdl resraw=rawr reschi=pearsonr;

**run**;

**1.6. SAS code for hybrid model**

Statistical model:

*logit(Result_pt_) = β_0_ + β_1_*Disease_pt_ +* ***β_2_*****PaperID_pt_ +* ***β_3_*****Disease*X_pt_ +* ***β_4_*****TestID_pt_ +* ***β_5_*****Disease_pt_*TestID_pt_*

Note we have dropped the *Disease_pt_*PaperID_pt_* interaction term, allowing the covariate to be estimated.

**PROC** **IMPORT** OUT= WORK.dvt

DATAFILE= "C:\Documents and Settings\mss4x\Desktop\data6

.xls"

DBMS=EXCEL2000 REPLACE;

GETNAMES=YES;

**RUN**;

**proc** **transreg** data=dvt design;

model class( paper gold setting test /deviations zero='1' '1' '1' '1' cprefix = **1** **4** **7 1**)

identity(prvlnc / tstandard=center name=(cprevalence))

bspline(fprc / degree=**3** nknots=**1** name=(bsfpr) )

;

id n result disease prvlnc cprvlnc;

output out=dvtno1;

**run**;

**proc** **genmod** data=dvtno1 ;

class paper test disease gold setting;

model result/n = disease

p2 p3 p4 p5 p6 p7 p8 p9 p10 p11 p12 p13 p14 p15 p16 p17 p18 p19 p20 p21 p22 p23

gold2

disease*gold2

setting2

disease*setting2

cprevalence

disease*cprevalence

t2 t3 t4 t5 t6 t7 t8 t9 t10 t11 t12 t13 t14 t15 t16 t17 t18 t19 t20 t21

disease*t2 disease*t3 disease*t4 disease*t5 disease*t6 disease*t7 disease*t8 disease*t9 disease*t10 disease*t11 disease*t12 disease*t13 disease*t14 disease*t15 disease*t16 disease*t17 disease*t18 disease*t19 disease*t20 disease*t21

/ dist=bin link=logit;

repeated subject=paper / within=test*disease type=exch covb corrw;

output out=dvtrsdl resraw=rawr reschi=pearsonr;

**run**;

**2. R codes**

**2.1. R code for plot for verifying Proportional Odds Ratio assumption graphically**

# remove almost all objects

rm(list=ls())

# read data file

dtfl <- read.table(file='g:/mir/byMSS/papers/PORforMetaAnalysisDiagnosticTests/caseStudy/obsVSfit.csv', sep=',', header=T)

str(dtfl)

### do not run this!

`data.frame': 66 obs. of 6 variables:

$ PAPER : Factor w/ 23 levels " 2Brenner, ..",..: 1 1 1 2 3 3 3 3 3 3 ...

$ TEST : Factor w/ 21 levels "Asserachrom..",..: 6 8 18 21 1 4 9 10 12 17 ...

$ OBSERVED: num 1.82 2.02 3.05 2.44 2.12 1.5 1.72 2.05 1.11 0.86 ...

$ FITTED : num 1.88 2.08 3.20 2.82 1.82 ...

$ pnmbr : int 2 2 2 4 5 5 5 5 5 5 ...

$ LABEL : Factor w/ 66 levels "Asserachrom..",..: 11 14 41 58 1 9 15 18 23 36 ...

###

# boxplot

plot(dtfl$PAPER, dtfl$OBSERVED)

# and its equivalent dotplot

plot(as.numeric(dtfl$PAPER), dtfl$OBSERVED) #, type='p')

# prepare paper and test IDs

pno <- as.numeric(dtfl$PAPER)

# mean paper LOR, for sorting x axis

paperLOR <- rep(NA, times=23)

for (i in 1:23) {

paperLOR[i] <- mean(dtfl$OBSERVED[pno==i])

NULL

}

#

ndx <- sort(paperLOR, index.return=T)

ndx <- ndx$ix

#

pnos <- pno

for (i in 1:23){

pnos[pno==ndx[i]] <- i

NULL

}

#

tno <- as.numeric(dtfl$TEST)

# per paper LOR (dotplot)

plot( 0,0, col='white', xlim=c(0,23), ylim=c(-.5, 5.5) , xlab='Paper No.' , ylab='Log Odds Ratio')

for (i in 1:length(ndx)) {

#points( rep(i, length=sum(pno==ndx[i])) , dtfl$OBSERVED[pno==ndx[i]] , pch=i) # , col=i

points( rep(i, length=sum(pnos==i)) , dtfl$OBSERVED[pnos==i] , pch=i) # , col=i

NULL

}

# plot for verifying Proportional Odds Ratio assumption graphicaly

table(as.numeric(table(tno))) # frequency of tests with certain number of readings.

lf <- 5/5 # lowess f span

mn <- 5 # minimum readigns for a test to be included.

tnos <- (1:21)[as.numeric(table(tno)) >=mn]

clrs <- c( "blue", "red", "purple", "green", "black")

#

plot( 0,0, col='white', xlim=c(0,23), ylim=c(0.7, 5.1) , xlab='Paper No.' , ylab='Observed Log-Odds-Ratio')

#for (i in 1:21) {

for (i in 1:length(tnos)) {

points( pnos[tno==tnos[i]] , dtfl$OBSERVED[tno==tnos[i]] , pch=i , col=clrs[i], cex=2 )

lines(lowess(pnos[tno==tnos[i]] , dtfl$OBSERVED[tno==tnos[i]], f=lf) , col=clrs[i], lty=i, lwd=3.5)

#lines( pnos[tno==tnos[i]] , dtfl$OBSERVED[tno==tnos[i]] , col=i)

NULL

}

legend(15,2, dimnames(table(dtfl$TEST))[[1]][tnos], col=clrs, pch=1:length(tnos), lty=1:length(tnos), lwd=2 )

**2.2. R code for observed-versus-fitted plot**

# GOF

dtfl <- read.table(file='G:/MIR/byMSS/papers/PORforMetaAnalysisDiagnosticTests/caseStudy/obsVSfit.csv', sep=',', header=T)

str(dtfl)

### do not run this!

`data.frame': 66 obs. of 6 variables:

$ PAPER : Factor w/ 23 levels " 2Brenner, ..",..: 1 1 1 2 3 3 3 3 3 3 ...

$ TEST : Factor w/ 21 levels "Asserachrom..",..: 6 8 18 21 1 4 9 10 12 17 ...

$ OBSERVED: num 1.82 2.02 3.05 2.44 2.12 1.5 1.72 2.05 1.11 0.86 ...

$ FITTED : num 1.88 2.08 3.20 2.82 1.82 ...

$ pnmbr : int 2 2 2 4 5 5 5 5 5 5 ...

$ LABEL : Factor w/ 66 levels "Asserachrom..",..: 11 14 41 58 1 9 15 18 23 36 ...

###

# simple observed-vs-fitted plot

tno <- as.numeric(dtfl$TEST)

plot(dtfl$OBSERVED, dtfl$FITTED, xlim=c(-.5,6.5), ylim=c(-.5,6.5) , xlab='Observed LOR', ylab='Fitted LOR')

lines(c(0,6), c(0, 6) , lty=2)

# observed-vs-fitted plot

plot(0,0, col='white', xlim=c(-.5,6.5), ylim=c(-.5,6.5) , xlab='Observed LOR', ylab='Fitted LOR')

for (i in 1:21) {

#points(dtfl$OBSERVED[tno==i], dtfl$FITTED[tno==i], pch=i , col=i )

#points(dtfl$OBSERVED[tno==i], dtfl$FITTED[tno==i], pch=i , col=1 , cex=2)

points(dtfl$OBSERVED[tno==i], dtfl$FITTED[tno==i], pch=i , col=i , cex=2) ; points(dtfl$OBSERVED[tno==i], dtfl$FITTED[tno==i], pch='*')

NULL

}

lines(c(0,6), c(0, 6) , lty=2)

lines(c(0,6-1), c(1, 6) , lty=2)

lines(c(0+1,6), c(0, 6-1) , lty=2)

legend(5,4.5, dimnames(table(dtfl$TEST))[[1]] , pch=1:21, col=1:21, cex=.91)

# observed-vs-fitted plot, with guidelines for % of change

# compute % change from obs to fit

prcnt <- abs((dtfl$OBSERVED - dtfl$FITTED)/dtfl$OBSERVED)

cbind(dtfl$OBSERVED, dtfl$FITTED, prcnt)

mean(prcnt); sd(prcnt)

hist(prcnt)

quantile(prcnt, c(.5,.9, .95, .97, .98, .99, 1))

# chose a 50% curve!

#

plot(0,0, col='white', xlim=c(-1.5,7), ylim=c(-1.5,7) , xlab='Observed LOR', ylab='Fitted LOR')

for (i in 1:21) {

points(dtfl$OBSERVED[tno==i], dtfl$FITTED[tno==i], pch=i , col=i , cex=2) ; points(dtfl$OBSERVED[tno==i], dtfl$FITTED[tno==i], pch='*')

NULL

}

lines(c(0,6), c(0, 6) , lty=2)

lines(c(0,6-1), c(1, 6) , lty=2)

lines(c(0+1,6), c(0, 6-1) , lty=2)

legend(5,4.85, dimnames(table(dtfl$TEST))[[1]] , pch=1:21, col=1:21, cex=.859)

#

x <- seq(-1, 5, length=100)

y <- x/2

lines(x, x+y, col=2)

lines(x, x-y, col=2)

**2.3. R code for plots of post-test probability differences, and NPVs**

### a pair of related performance measures, like the following, that depend on prevalence too!

### graph of: pre-test vs post-test prob(Disease | normal vs abnormal test result), and the difference curve.

### defining a function for drawing positive and negative predictive curves for a diagnostic odds ratio DOR:

# do not need upper x axis rug if rug=T

dor.pv <- function(dor,

prevalence=NULL, sensitivity=NULL, specificity=NULL,

xprevalence='specificity',

npoints=100, plot=T, rug=F, legend=T) {

# FUNCTION NAME:

# dor.pv

# DESCRIPTION:

# drawing positive and negative predictive curves for a diagnostic odds ratio DOR.

# USAGE:

# dor.pv(dor,

# prevalence=NULL, sensitivity=NULL, specificity=NULL,

# xprevalence='specificity',

# npoints=100, plot=T, rug=F, legend=T)

#

# ARGUMENTS:

# dor: diagnostic odds ratio.

# prevalence: in 0-1 scale.

# sensitivity: in 0-1 scale.

# specificity: in 0-1 scale. it needn't to specify both the sensitivity and specificity.

# xprevalence: a character string, for when the prevalence is inputted, there are two choices of the x-axis in the graph: either 'sensitivity' or 'specificity' (default).

# npoints: number of points where coordinates of curve are computed. The bigger the npoints, the more accurate the curve.

# plot: a logical, whether to plot the curve.

# rug: a logical, whether to add rugs to the curve, defaults to False. It is a good idea to set rug to True in the draft version of graph, to make sure there are enough points estimated across all regions of the curve. For large npoints, setting rug to F reduces running time.

# legend: a logical, whether to add legend to the graph.

# VALUE:

# returns, invisbly, a list of three components.

# the first component is a matrix of 4 columns and npoints rows,

# where the columns are specificity, ppv, sensitivity, and npv.

# the second component is a matrix of 3 columns and npoints rows,

# where the columns are prevalence, ppv, and npv.

# the third component contains ppv and npv only when both prevalence and sensitivity (or specificity) are inputted.

# if none of the prevalence or sensitivity (or specificity) are inputted, it returns a list of 5 components,

# prevalence, tpr, ppv, fpr, and npv. the ppv and npv are matrices that define the z coordinates of the 3D plots.

# DETAILS:

# if only DOR is inputted, two 3D plots are produced. hence a surface is computed for ppv, and for npv.

# if either prevalence or sensitivity (equivalently specificty) is specified, one plot is produced. hence a curve is computed for ppv, and for npv.

# if both prevalence or sensitivity (equivalently specificty) are specified, one the curves a point estimate for ppv and for npv are computed on the curves.

#

# EXAMPLES:

# > # assuming dor=45.3, prevalence=0.23 (23%), and sensitivity=.932 (93.2%)

# > dor.pv(45.3, pre=.23, sens=.932)

# giving spec rather than sens

# > # the same graph, with rugs

# > dor.pv(45.3, pre=.23, sens=.932, rug=T)

# > # the same numbers, but asking for sensitivity to be the x-axis for the first graph

# > dor.pv(45.3, pre=.23, sens=.932, xpre='sensitivity')

# > # assuming sensitivity is not known, hence only dor and prevalence.

# > dor.pv(45.3, pre=.23)

# > # assuming prevalence is not known, hence only dor and sensitivity.

# > dor.pv(45.3, sens=.932)

# > # assuming only dor is known.

# > dor.pv(45.3)

#

# DEPENDENCIES:

# tested for R 1.7.0 .

# AUTHOR:

# mir drdresearch@yahoo.com & http://www.people.virginia.edu/~mss4x/

# last updated 2august03

# REFERENCE:

# checking

if ( !is.null(sensitivity) & !is.null(specificity) ) {

spcfcty <- dor*(1-sensitivity)/sensitivity

spcfcty <- spcfcty/(1+spcfcty)

if ( round(specificity,2) != round(spcfcty,2) ) stop('Sensitivity and Specificity do not match the DOR!')

# title( main=list(paste('point estimates: PPV', round(ppv[ndx],2) ,'and NPV', round(npv[ndx], 2) ) ))#, adj=0))

}

# compute sens based on spec.

if ( is.null(sensitivity) & !is.null(specificity) ) {

sensitivity <- dor*(1-specificity)/specificity

sensitivity <- sensitivity/(1+sensitivity)

}

# initiate graphics device, one graph/page.

if ( !xor(is.null(prevalence), is.null(sensitivity)) ) par(mfrow=c(1,2)) else {par(mfrow=c(1,1)) ; par(omd=c(0,0,0,0)) }

# initiate result object

result <- list(prevalenceFixed=NA, sensitivityFixed=NA, summary=NA)

point.ppv <- point.npv <- NULL

# when prevalence is given.

if (!is.null(prevalence)) {

# making an arbitrary sequence (x coordinate).

tpr <- seq(0,1, length=npoints)

# computing y coordinate.

ppv <- 1/ (1+ ((1-prevalence)/prevalence) * (1/ (dor-tpr*(dor-1))) )

# computing alternate x coordiante.

tmp <- dor*(1-tpr)/tpr

fpr <- 1/(1+tmp)

# npv

npv <- 1/ (1+ (prevalence/(1-prevalence)) * (1/ (dor-(1-fpr)*(dor-1))) )

# plot.

if (plot) {

# ppv

if (xprevalence=='specificity') x <- 1-fpr else x <- tpr

plot(x , ppv, type='l', lty=1, col='black', xlab=xprevalence, ylab='Predictive Values', ylim=c(0,1))

# add on npv

lines( x , npv, lty=2, col='red', lwd=2)

if (xprevalence=='specificity') tmp <- 'sensitivity' else tmp <- 'specificity'

title(main=list(tmp, col='black', cex=1, font=1))

# second (upper) horizontal axis, for tpr.

nticks <- 11 # number of tick marks for the tpr-axis.

ndx <- seq(1, length(tpr), length=nticks)

lbls <- tpr[ndx] # labels for tpr-axis.

lbls <- round(lbls, 1) # one decimal digit only.

pstn <- (1-fpr)[ndx] # positions of labels for tpr-axis.

axis(3, at=pstn, labels=lbls, col='black', col.axis='black')

# subtitle

title( sub=list( paste('For DOR of', dor, 'and Prevalence of', round(prevalence*100, 1), '%' ) , cex=.75), adj=0)

# rugs

if (rug) {

rug( (1-fpr), side=1)

rug(ppv, side=2)

# for npv

rug( (1-fpr), side=3, col='red', ticksize=.02)

rug(npv, side=2, col='red', ticksize=.02)

} # end rug.

# legend.

if (legend) legend(.05, .6, c('PPV', 'NPV'), col=c('black', 'red'), lty=c(1,2), lwd=c(1,2))

}

result[[1]] <- cbind(specificity=(1-fpr), ppv, sensitivity=tpr, npv)

} # end plot.

# when sensitivity is given.

if (!is.null(sensitivity)) {

# computing specificity, based on inputted sens and dor.

spcfcty <- dor*(1-sensitivity)/sensitivity

spcfcty <- spcfcty/(1+spcfcty)

# add observed points to the previous plot, if any.

if (!is.null(prevalence)) {

if (xprevalence=='specificity') rx <- spcfcty else rx <- sensitivity

rug(rx, side=1, ticksize=.04, col='blue') # on x-axis

rug(rx, side=3, ticksize=.04, col='blue') # on x-axis 2

# for ppv

ndx <- abs(x-rx)==min(abs(x-rx)) # find closest point.

point.ppv <- ppv[ndx] # point estimate for ppv.

rug(point.ppv, side=2, ticksize=.04, col='blue')

points(rx, point.ppv, col='blue', pch=19)

# for npv

point.npv <- npv[ndx] # point estimate for npv.

rug(point.npv, side=2, ticksize=.04, col='blue')

points(rx, point.npv, col='blue', pch=19)

# add to result

result[[3]] <- cbind(point.ppv, point.npv)

}

# making an arbitrary sequence (x coordinate).

prvlnc <- seq(0,1, length=npoints)

# computing y coordinate.

ppv <- 1/ (1+ ((1-prvlnc)/prvlnc) * (1/ (dor-sensitivity*(dor-1))) )

# npv

npv <- 1/ (1+ (prvlnc/(1-prvlnc)) * (1/ (dor-spcfcty*(dor-1))) )

# plot.

if (plot) {

# ppv

plot(prvlnc , ppv, type='l', lty=1, col='black', xlab='prevalence', ylab='Predictive Values', ylim=c(0,1))

# add on npv

lines(prvlnc, npv, lty=2, col='red', lwd=2)

# subtitle

title( sub=list( paste('For DOR of', dor, ', Sensitivity of', round(sensitivity*100, 1), '%', '(or Specificity of', round(spcfcty*100, 1), '%)' ) , cex=.75), adj=0)

# rugs

if (rug) {

rug(prvlnc, side=1)

rug(ppv, side=2)

# for npv

rug(prvlnc, side=3, col='red', ticksize=.02)

rug(npv, side=2, col='red', ticksize=.02)

} # end rug.

# legend.

if (legend & is.null(prevalence)) legend(.05, .6, c('PPV', 'NPV'), col=c('black', 'red'), lty=c(1,2), lwd=c(1,2))

# add observed points to the plot, if any.

if (!is.null(prevalence)) {

rx <- prevalence

rug(rx, side=1, ticksize=.04, col='blue') # on x-axis

# for ppv

x <- prvlnc

ndx <- abs(x-rx)==min(abs(x-rx)) # find closest point. # this calculation is redundant/for safety.

rug(ppv[ndx], side=2, ticksize=.04, col='blue')

points(rx, ppv[ndx], col='blue', pch=19)

# for npv

rug(npv[ndx], side=2, ticksize=.04, col='blue')

points(rx, npv[ndx], col='blue', pch=19)

}

} # end plot.

result[[2]] <- cbind(prvlnc, ppv, npv)

}

# if only dor inputted, make a 3D graph.

if (is.null(prevalence) & is.null(sensitivity)) {

npoints <- round(npoints/5)

tpr <- seq(0,1, length=npoints)

prvlnc <- seq(0,1, length=npoints)

# for ppv

f <- function(prevalence, tpr, dor) { r <- 1/ (1+ ((1-prevalence)/prevalence) * (1/ (dor-tpr*(dor-1))) ) }

ppv <- outer(prvlnc, tpr, FUN=f, dor)

# plot.

if (plot) persp(prvlnc, tpr, ppv, theta=-130, phi=20, expand=.5 , ticktype='detailed', col='lightblue', shade=.7, xlab='Prevalence', ylab='Sensitivity')

# for npv

tmp <- dor*(1-tpr)/tpr

fpr <- 1/(1+tmp)

f <- function(prevalence, fpr, dor) { r <- 1/ (1+ (prevalence/(1-prevalence)) * (1/ (dor-(1-fpr)*(dor-1))) ) }

npv <- outer(prvlnc, fpr, FUN=f, dor)

# plot.

if (plot) persp(prvlnc, fpr, npv, theta=60, phi=20, expand=.5 , ticktype='detailed', col='lightblue', shade=.5, xlab='Prevalence', ylab='1-Specificity')

# return

result <- list(prvlnc, tpr, ppv, fpr, npv)

return(invisible(result))

}

# back the graphics.

par(mfrow=c(1,1))

if ( !is.null(sensitivity) & !is.null(prevalence) ) {

# add text for ppv and npv.

par(omd=c(0,0,.05,0)) # increase outer margin for the title.

title( main=list(paste('point estimates: PPV', round(ppv[ndx],2) ,'and NPV', round(npv[ndx], 2) ) ) , outer=T )

#, adj=0), outer=T)

}

# back margin.

par(omd=c(0,0,0,0))

# return invisibly.

invisible(result)

}

### END function dor.pv .

## plot of PPV&NPV for diagnostic test SimpliRED

t1 <- 'SimpliRED'

t1.dor <- exp(2.6925) # SimpliRED for single test case

t1.sens <- .84

t1.prvlnc <- .28

#

tmp <- dor.pv(dor=t1.dor , sens=t1.sens)

## define parameters

t1 <- 'VIDAS'

t2 <- 'D-Dimer'

t1.dor <- exp(2.489 + 1.004) # VIDAS fitted by model

t2.dor <- exp(2.489 + .225)

t1.sens <- .97 #median observed unweighted

t2.sens <- .73 # D-dimer Test

t1.prvlnc <- .44 # for graphing NOT computations

t2.prvlnc <- .44

## plot for post-test probability difference (for VIDAS)

tmp <- dor.pv(dor=t1.dor , sens=t1.sens)

#

npv.t1 <- tmp$sensitivityFixed[,3]

prvlnc <- tmp$sensitivityFixed[,1]

pnr.t1 <- 1- npv.t1 # post test probability of normal test.

pab.t1 <- tmp$sensitivityFixed[,2] # post test probability of abnormal test (=ppv).

#

## single test graph

plot(prvlnc, pnr.t1, type='l', col=2, lty=2, ylab='Post-Test Probability of Disease' , xlab='Pre-Test Probability of Disease (prevalence)', ylim=c(0,1), lwd=2) # normal Test Result

lines(prvlnc, pab.t1, type='l', col=4, lty=4, lwd=2) # Abnormal Test Result

lines(c(0,1), c(0,1), lty=3, col=3 , lwd=1)# line of pure chance, uninformative test

#legend(.15,.82, c('Normal Test Result','Abnormal Test Result', 'non-informative'), col=1:3, lty=1:3, lwd=2 )

lines(prvlnc, pab.t1 - pnr.t1 , type='l', col=1, lty=1, lwd=1) # post test prob difference

legend(.05,.95, c('Normal Test Result','Abnormal Test Result', 'uninformative', 'Post Test Probability Difference'), col=c(2,4,3,1), lty=c(2,4,3,1), lwd=2 )

#

# to find maximum of difference curve, and the prevalence

prvlnc[max(pab.t1 - pnr.t1) == (pab.t1 - pnr.t1)]; max(pab.t1 - pnr.t1)

# to find normal and abnormal post values for a prevalnce of 40%, for example

cbind(prvlnc, pnr.t1, pab.t1)

## plot comparing post-test probability difference of two diagnostic tests (VIDAS versus D-Dimer).

tmp <- dor.pv(dor=t2.dor , sens=t2.sens )

npv.t2 <- tmp$sensitivityFixed[,3]

pnr.t2 <- 1- npv.t2 # post test probability of normal test.

pab.t2 <- tmp$sensitivityFixed[,2] # post test probability of abnormal test (=ppv).

#

# 2 difference curves together

delta1 <- pab.t1 - pnr.t1

delta2 <- pab.t2 - pnr.t2

plot(prvlnc, delta1 , type='l', col=2, lty=2, ylab='Post-Test Probability Difference' , xlab='Pre-Test Probability of Disease (prevalence)', ylim=c(min(c(delta1-delta2,0)),1), lwd=2) # difference curve for test1

lines(prvlnc, delta2, type='l', col=4, lty=4, lwd=2) # difference curve for test2

lines(prvlnc, delta1-delta2, type='l', col=1, lty=1, lwd=1) # difference of two tests

abline(h=0, lty=3, col=3)

legend(.1,.9, c(t1, t2, 'indifference', paste( t1, ' - ', t2, sep='')), col=c(2,4,3,1), lty=c(2,4,3,1), lwd=2 )

#

# finding optima

prvlnc[min(delta1-delta2) == (delta1-delta2)]; min(delta1-delta2)

prvlnc[max(delta1-delta2) == (delta1-delta2)]; max(delta1-delta2)

### comparing diagnostic tests based on their NPV (or other measures that depend on prevalence), so to find at what prevalence the difference between NPV of the competing tests is maximized, or minimized.

## generic

t1 <- 'VIDAS'

t2 <- 'D-Dimer'

t1.dor <- exp(2.489 + 1.004) # VIDAS fitted by model

t2.dor <- exp(2.489 + .225)

t1.sens <- .97 #median observed unweighted

t2.sens <- .73 # D-dimer Test

t1.prvlnc <- .44 # for graphing NOT computations

t2.prvlnc <- .44

#

tmp <- dor.pv(dor=t1.dor , sens=t1.sens)

npv.t1 <- tmp$sensitivityFixed[,3]

prvlnc <- tmp$sensitivityFixed[,1]

#

tmp <- dor.pv(dor=t2.dor , sens=t2.sens )

npv.t2 <- tmp$sensitivityFixed[,3]

## plot of the 3 curves together

plot(prvlnc, npv.t1 - npv.t2, type='l', col=1, ylab='NPV' , xlab='prevalence', ylim=c(0,1))

lines(prvlnc, npv.t1, type='l', col=2, lty=2, lwd=2)

lines(prvlnc, npv.t2, col=4, lty=4, lwd=2)

title(main='Difference in "Negative Predictive Values"')

rug( prvlnc[(npv.t1 - npv.t2) == max(npv.t1 - npv.t2)], col=2 )

rug( c(t1.prvlnc, t1.prvlnc), col=1 )

lines(c(0,1), c(1,0), lty=3, col=3 , lwd=1)# line of pure chance, uninformative test

legend(.1,.49, c(t1, t2, 'indifference', paste( t1, ' - ', t2, sep='')), col=c(2,4,3,1), lty=c(2,4,3,1), lwd=2 )

#

prvlnc[(npv.t1 - npv.t2) == max(npv.t1 - npv.t2)] ; max(npv.t1 - npv.t2)

**2.4. R code for heterogeneous ROC curve**

#### plot heterogeneous ROC curve (HetROC) .

dtfl <- read.table(file='g:/Mir/bymss/papers/PORforMetaAnalysisDiagnosticTests/caseStudy/dataNewExcerpt2.csv', header=T, sep=',')

str(dtfl)

### do no run this!

`data.frame': 65 obs. of 26 variables:

$ PAPER : int 2 2 2 4 5 5 5 5 5 5 ...

$ AUTHOR : Factor w/ 19 levels "Brenner, B.",..: 1 1 1 3 4 4 4 4 4 4 ...

$ YEAR : int 1995 1995 1995 1996 1996 1996 1996 1996 1996 1996 ...

$ JOURNAL : Factor w/ 13 levels "Acta Clinic..",..: 4 4 4 12 12 12 12 12 12 12 ...

$ REFERENC: Factor w/ 21 levels "1995;6:219-..",..: 1 1 1 3 4 4 4 4 4 4 ...

$ COUNTRY : Factor w/ 11 levels "Belgium","Canada",..: 4 4 4 5 3 3 3 3 3 3 ...

$ TEST : Factor w/ 21 levels "Asserachrom",..: 6 8 18 21 1 4 9 10 12 17 ...

$ MANUFACT: Factor w/ 10 levels "Agen","bioMerieux",..: 1 1 1 2 10 10 4 8 10 7 ...

$ CUTOFF : Factor w/ 14 levels ";NULL!","0.25",..: 12 12 1 12 12 12 9 8 12 12 ...

$ SENS.T : num 87 80 94 96 97 73 93 93 93 80 ...

$ SPEC.T : num 48 67 61 44 22 62 30 37 19 38 ...

$ TEST2 : int 7 9 20 23 2 5 10 12 14 19 ...

$ SUSPDVT : int 86 86 86 103 171 171 171 171 171 171 ...

$ NDVT : int 50 50 50 22 75 75 75 75 75 75 ...

$ NNODVT : int 36 36 36 81 96 96 96 96 96 96 ...

$ TP : int 44 40 47 21 73 55 70 70 70 60 ...

$ FN : int 6 10 3 1 2 20 5 5 5 15 ...

$ TN : int 17 24 22 36 21 60 29 36 18 36 ...

$ FP : int 19 12 14 45 75 36 67 60 78 60 ...

$ PRVLNC : int 58 58 58 21 44 44 44 44 44 44 ...

$ SETTING : Factor w/ 2 levels "In-Mix","Outpat..": 2 2 2 1 1 1 1 1 1 1 ...

$ SETTING2: int 3 3 3 2 2 2 2 2 2 2 ...

$ GOLD.STD: Factor w/ 9 levels "US","US compres..",..: 4 4 4 2 4 4 4 4 4 4 ...

$ GOLD : Factor w/ 2 levels "US-V","V": 1 1 1 1 1 1 1 1 1 1 ...

$ GOLD2 : int 1 1 1 1 1 1 1 1 1 1 ...

$ LABEL : Factor w/ 21 levels " 2Brenner, ..",..: 1 1 1 2 3 3 3 3 3 3 ...

###

# FPR

dtfl$fpr <- 1- (dtfl$SPEC.T/100)

# get list of tests

tmp <- aggregate(dtfl$TEST, by= list(dtfl$TEST), FUN=length )

id <- tmp[,1]

library(splines)

## test id

it <- 18 #21

npoints <- 100

dfseq <- 1:4

spln <- T

#

tmp <- dtfl[dtfl$TEST == id[it] ,]

tpr <- tmp$SENS.T/100

tpr[tpr ==1] <- .99999 # change 100% sens

fpr <- tmp$fpr

z <- log((tpr*fpr)/((1-tpr)*(1-fpr)))

z <- c(z,z) # because of grouped binary structure

# this is for the text() for observed points.

ss <- tmp$TP + tmp$FN + tmp$TN + tmp$FP

# make grouped binary response matrix

tmp <- rbind( cbind(tmp$TP, tmp$FN, d=1), cbind(tmp$FP, tmp$TN, d=0) )

d <- tmp[,3]

fpr2 <- seq(0,1, length=npoints)

#

ft.hm <- glm( tmp[,1:2] ~ d , family=binomial)

tpr.hm <- 1/(1+(1/(exp(ft.hm$coefficients[2])*fpr2/(1-fpr2))))

## fit natural spline

# homo ROC

plot(fpr2, tpr.hm, type='l', ylim=c(0,1), xlim=c(0,1), xlab='False Positive Rate', ylab='True Positive Rate', lty=2, col=1)

#

z <- c(fpr,fpr) # because of grouped binary structure

#

dfseq <- 2#3# 1:3

#

# initiate object to capture all spline fitted, and their TPR

fts.spln <- as.list(rep(NA, length(dfseq)))

tpr.spln <- as.list(rep(NA, length(dfseq)))

for (i in (1:length(dfseq))) {

dgfr <- dfseq[i]

fts.spln[[i]] <- glm( tmp[,1:2] ~ d + ns(z, dgfr) + d*ns(z, dgfr) , family=binomial)

d2 <- rep(1, times=length(fpr2)) # prediction under D+ gives values for odds TP/FN

lotpfn <- predict(fts.spln[[i]], newdata=as.data.frame(cbind(d=d2, z=fpr2)) ) # log-odds of T+/T- when D+

#lotpfn <- safe.predict.gam(ft.spln, newdata=as.data.frame(cbind(d=d2, z=fpr2)) ) # log-odds of T+/T- when D+

d2 <- rep(0, times=length(fpr2)) # prediction under D- gives values for odds FP/TN

lofptn <- predict(fts.spln[[i]], newdata=as.data.frame(cbind(d=d2, z=fpr2)) ) # log-odds of T+/T- when D+

lor <- lotpfn - lofptn

tpr.spln[[i]] <- 1/(1+(1/( exp(lor)*fpr2/(1-fpr2))))

lines(fpr2, tpr.spln[[i]], col=i+1, lty=i, lwd=2)

}

lgnd <- c('Homogeneous ROC', paste('Heterogeneous ROC with Spline DF =' , dfseq ) )

lgndcol <- c(1,2) # c(1, dfseq+1)

lgndlty <- 2:1

legend(.35,.4, lgnd, col=lgndcol, lwd=2 , lty=lgndlty , cex=.9)

# add observed points; size proportional to sample size of PaperID (study).

scl <- 1/2 # to scale the size of points proportionatel

for (i in 1:length(fpr)) {

#points(fpr[i], tpr[i], cex=ss[i]*scl)

points(fpr[i], tpr[i], cex=sqrt(ss[i])*scl) # area of circle is proportional to sampsi

NULL

}

# this adds text sample size

# observed points

#points(fpr, tpr , pch=15)

#text(fpr, tpr, ss, pos=1, cex=.6)

#

#par(mfrow=c(1,1))

#title(main=as.character(id[it]))

**2.5. R code for Funnel plot, Radial plot, and L’Abbe plot**

### making funnel plot, radial, L'Abbe, etc. !

# page 113 Sutton

dtfl <- read.table(file='g:/mir/byMSS/papers/PORforMetaAnalysisDiagnosticTests/caseStudy/data6_lor2.csv', sep=',', header=T)

str(dtfl)

### do not run this!

`data.frame': 66 obs. of 20 variables:

$ PAPER : int 1 1 1 2 3 3 3 3 3 3 ...

$ PAPERLAB: Factor w/ 23 levels " 2Brenner, ..",..: 1 1 1 2 3 3 3 3 3 3 ...

$ GOLD : int 1 1 1 1 1 1 1 1 1 1 ...

$ GOLDLABE: Factor w/ 2 levels "US-V","V": 1 1 1 1 1 1 1 1 1 1 ...

$ SETTING : int 2 2 2 1 1 1 1 1 1 1 ...

$ SETTINGL: Factor w/ 2 levels "In-Mix","Outpat..": 2 2 2 1 1 1 1 1 1 1 ...

$ PRVLNC : int 58 58 58 21 44 44 44 44 44 44 ...

$ CPRVLNC : int 20 20 20 -17 6 6 6 6 6 6 ...

$ TEST : int 6 8 18 21 1 4 9 10 12 17 ...

$ TESTLABE: Factor w/ 21 levels "Asserachrom",..: 6 8 18 21 1 4 9 10 12 17 ...

$ OBSERVED: num 1.82 2.02 3.05 2.44 2.12 ...

$ SELORC : num 0.534 0.491 0.648 0.873 0.688 ...

$ FPRC : num 0.53 0.34 0.39 0.55 0.78 0.38 0.7 0.62 0.81 0.62 ...

$ TPRC : num 0.87 0.79 0.93 0.93 0.97 0.73 0.93 0.93 0.93 0.8 ...

$ FITTED : num 1.88 2.08 3.20 2.82 1.82 ...

$ PNMBR : int 2 2 2 4 5 5 5 5 5 5 ...

$ LABEL : Factor w/ 66 levels "Asserachrom..",..: 11 14 41 58 1 9 15 18 23 36 ...

$ FITTPRC : num 0.881 0.805 0.940 0.954 0.956 ...

$ TPRC2 : num 0.874 0.796 0.931 0.934 0.967 ...

$ CFPRC : num 0.09 -0.1 -0.05 0.11 0.34 -0.06 0.26 0.18 0.37 0.18 ...

###

### funnel plot, for publication bias

plot(dtfl$OBSERVED, 1/dtfl$SELORC, xlab='Log Odds Ratio', ylab='1 / SE(LOR)', pch=dtfl$TEST, cex=2)

abline( v = sum( dtfl$OBSERVED * (1/(dtfl$SELORC * sum(1/dtfl$SELORC))) ) , col=3, lty=1) # weighted average

#abline( v = mean(dtfl$OBSERVED) , col=4, lty=2) # unweighted average

#

# using color

plot(dtfl$OBSERVED, 1/dtfl$SELORC, xlab='Log Odds Ratio', ylab='1 / SE(LOR)', pch=dtfl$TEST, cex=2, col=dtfl$TEST ) #1:4)

points(dtfl$OBSERVED, 1/dtfl$SELORC, col=1 ) #, pch='.')

abline( v = sum( dtfl$OBSERVED * (1/(dtfl$SELORC * sum(1/dtfl$SELORC))) ) , col=3, lty=1) # weighted average

### radial plot, for contribution to heterogeneity

plot(1/dtfl$SELORC, dtfl$OBSERVED/dtfl$SELORC , xlab='1 / SE(LOR)', ylab='Log Odds Ratio / SE(LOR)', pch=dtfl$TEST, cex=2, col=dtfl$TEST )

points(1/dtfl$SELORC, dtfl$OBSERVED/dtfl$SELORC, col=1 )

y <- dtfl$OBSERVED/dtfl$SELORC

x <- 1/dtfl$SELORC

#tmp <- lm( y ~ x ); summary(tmp); abline(a=tmp$coefficients[1], b=tmp$coefficients[2])

tmp <- lm( y ~ x -1 ) ; summary(tmp)

abline(a=0, b=tmp$coefficients[1], col=4)

abline(a=sqrt(sum(tmp$residuals^2)/tmp$df.residual) , b=tmp$coefficients[1], col=1, lty=2)

abline(a=-sqrt(sum(tmp$residuals^2)/tmp$df.residual) , b=tmp$coefficients[1], col=1, lty=2)

### L'Abbe plot, for heterogeneity

x <- dtfl$FPRC/ (1-dtfl$FPRC)

y <- dtfl$TPRC/ (1-dtfl$TPRC)

plot(x,y, col='white', xlab='Odds of Positive Test Result in the Healthy', ylab='Odds of Positive Test Result in the Sick')

#points(x,y, cex=1/(dtfl$SELORC*1)) # radius is proportional to 1/SE

points(x,y, cex=1/(sqrt(dtfl$SELORC/pi)*.51)) # area of each point is proportional to 1/SE

#

tmp <- lm( y ~ x ); summary(tmp); log(tmp$coefficients[2]); abline(a=tmp$coefficients[1], b=tmp$coefficients[2])

### this plot may not be suitable for this outcome!!!!

plot(0,0, col='white', xlim=c(0,1), ylim=c(0,1))

points(dtfl$FPRC, dtfl$TPRC, cex=1/(dtfl$SELORC*1))

## logit tranform, to straighten

logit <- function(x) { # assuming 0<x<1

log(x/(1-x))

}

#

#plot(0,0, col='white', xlim=c(-5,5), ylim=c(-5,5))

plot(0,0, col='white', xlim=c(-3,2), ylim=c(-1,5), xlab='logit(FPR)', ylab='logit(TPR)')

#points(logit(dtfl$FPRC), logit(dtfl$TPRC), cex=1/(dtfl$SELORC*1), col=dtfl$TEST)

#points(logit(dtfl$FPRC), logit(dtfl$TPRC), cex=.1, col=1)

points(logit(dtfl$FPRC), logit(dtfl$TPRC), cex=1/(dtfl$SELORC*1), pch=dtfl$TEST)

**2.6. R code for converting DOR to other performance measures**

### converting DOR to other performance measures

# code taken from META paper of JCE 2004

#source('G:/MIR/byMSS/papers/PORforMetaAnalysisDiagnosticTests/caseStudy/dor.convert_2.txt')

## define function

dor.convert <- function(dor, prevalence, sensitivity=NULL, specificity=NULL) {

# ARGUMENTS:

# dor: a numeric vector.

# prevalence

# sensitivity or specificity: either one should be specified.

# VALUE:

# ppv

# npv

# lrab

# lrnr

# preodds

# postoddsab

# postoddsnr

# postpab

# postpnr

# auc

#

# EXAMPLES:

# dor.convert(dor=34, pre=.2, sen=.9)

# dor.convert(dor=c(34, 25, 1.5), pre=.2, sen=.9)

# DEPENDENCIES:

# tested for R 1.7.0 .

# AUTHOR:

# mir drdresearch@yahoo.com & http://www.people.virginia.edu/~mss4x/

# last updated 3august03

# REFERENCE:

# http://www.cebm.net/likelihood_ratios.asp , accessed aug03

# checking

if ( !is.null(sensitivity) & !is.null(specificity) ) {

spcfcty <- dor*(1-sensitivity)/sensitivity

spcfcty <- spcfcty/(1+spcfcty)

if ( round(specificity,2) != round(spcfcty,2) ) stop('Sensitivity and Specificity do not match the DOR!')

}

# compute sens based on spec.

if ( is.null(sensitivity) & !is.null(specificity) ) {

sensitivity <- dor*(1-specificity)/specificity

sensitivity <- sensitivity/(1+sensitivity)

}

# compute spec based on sens.

if ( !is.null(sensitivity) & is.null(specificity) ) {

specificity <- dor*(1-sensitivity)/sensitivity

specificity <- specificity/(1+specificity)

}

# ppv

ppv <- 1/ (1+ ((1-prevalence)/prevalence) * (1/ (dor-sensitivity*(dor-1))) )

# npv

npv <- 1/ (1+ (prevalence/(1-prevalence)) * (1/ (dor-specificity*(dor-1))) )

# lrab

lrab <- sensitivity/(1-specificity)

# lrnr

lrnr <- (1-sensitivity)/specificity

# preodds

preodds <- prevalence/(1-prevalence)

# postoddsab

postoddsab <- preodds * lrab

# postoddsnr

postoddsnr <- preodds * lrnr

# postpab

postpab <- postoddsab/(postoddsab+1)

# postpnr

postpnr <- postoddsnr/(postoddsnr+1)

# homogeneous AUC

# based on Walter SD. Properties of the summary receiver operating characteristic (SROC) curve for diagnostic test data. Stat Med. 2002 May 15; 21(9):1237-56. PMID: 12111876

h.auc <- (dor*(dor-1-log(dor))) / ((dor-1)^2)

# pack the results

inputs <- rbind(DiagnosticOddsRatio=dor, prevalence, sensitivity, specificity)

outputs <- rbind(h.auc, PositivePredictiveValue=ppv, NegativePredictiveValue=npv, LikelihoodRatioForAbnormalTest=lrab, LikelihoodRatioForNormalTest=lrnr, PreTestOdds=preodds, PostTestOddsOfAbnormalTest=postoddsab, PostTestOddsOfNormalTest=postoddsnr, PostTestProbabilityOfAbnormalTest=postpab, PostTestProbabilityOfNormalTest=postpnr)

result <- list(inputs, outputs)

}

## END define function

# parameter estimates, from the modeling

dor <- c(

20.34835351,

15.04882628,

4.463351762,

15.08498684,

1.488099794,

4.758345387,

9.939333837,

5.801984418,

17.95197255,

28.37759712,

27.04494198,

21.05840989,

10.44162269,

14.45730756,

8.721576388,

23.16169879,

5.430330513,

17.85350772,

24.33705291,

8.678077344,

32.87458938

)

tmp <- dor.convert(dor=dor, prevalence=.4, sensitivity=.9, specificity=NULL)

tmp[[1]]

tmp[[2]]

# we fix specificity, since these tests are mainly used for ruling out

tmp <- dor.convert(dor=dor, prevalence=.4, sensitivity=NULL, specificity=.9 )

tmp[[1]]

tmp[[2]]

# save to disk, to export to Excel

write.table( t(tmp[[1]]), file='G:/MIR/byMSS/papers/PORforMetaAnalysisDiagnosticTests/caseStudy/dor.translated.1.csv', sep=',', row.names=F)

write.table( t(tmp[[2]]), file='G:/MIR/byMSS/papers/PORforMetaAnalysisDiagnosticTests/caseStudy/dor.translated.2.csv', sep=',', row.names=F)
